# Taming the Immiscibility of Gold, Iron, and Boron to Craft Chemodegradable Nanoparticles for Multimodal Imaging and Radiotherapy

**DOI:** 10.1002/adhm.202505031

**Published:** 2026-02-15

**Authors:** Michael Bissoli, Clara M.G. de Faria, Veronica Torresan, Maria Assunta Lacavalla, Mattia Cattelan, Denis Badocco, Paolo Pastore, Pasquina Marzola, Laura Cansolino, Cinzia Ferrari, Ian Postuma, Riccardo Vago, Silva Bortolussi, Antonello E. Spinelli, Daniel Forrer, Vincenzo Amendola

**Affiliations:** ^1^ Department of Chemical Sciences University of Padova Padova Italy; ^2^ Department of Engineering for Innovation Medicine University of Verona Verona Italy; ^3^ Department of Clinical Surgical Sciences Integrated Unit of Experimental Surgery Advanced Microsurgery and Regenerative Medicine University of Pavia Pavia Italy; ^4^ INFN (National Institute of Nuclear Physics), Unit of Pavia Pavia Italy; ^5^ Urological Research Institute Division of Experimental Oncology IRCCS San Raffaele Scientific Institute Milan Italy; ^6^ Department of Physics University of Pavia Pavia Italy; ^7^ Experimental Imaging Center IRCCS San Raffaele Scientific Institute Milan Italy; ^8^ CNR – ICMATE Padova Italy

**Keywords:** DFT, imaging, laser ablation, nanoalloys, radiotherapy

## Abstract

Despite more than half of all oncological patients undergo X‐ray radiotherapy (XRT), significant efforts are required to improve its efficacy against hypoxic tumor regions and, at the same time, to expand the therapeutic window to spare normal tissues. The use of radiosensitizers, the personalization of radiation dose planning aided by imaging with magnetic resonance imaging (MRI) and X‐ray computed tomography (CT), and the implementation of boron neutron capture therapy (BNCT) are three strategies to encompass the limits of XRT. Here, these three strategies are leveraged by designing and achieving a theranostic platform based on trimetallic Au‐Fe‐B nanoparticles (NPs). According to density functional theory calculations, chemodegradable Au‐Fe‐B nanostructures are not achievable under thermodynamic equilibrium conditions. Hence, Au‐Fe‐B NPs were synthesized by laser ablation in liquid, because it is a nonequilibrium process, followed by a tailored cleaning protocol. The Au‐Fe‐B NPs were coated with biocompatible polymers and showed several useful properties for nanomedicine application, such as chemical degradation in a physiological environment, contrast ability for MRI and CT, in vitro radiosensitization efficacy for XRT and BNCT, and consistent intracellular uptake. These functionalities can enable advanced studies on tumor treatment with complementary therapeutic strategies guided by anatomic imaging, leading to more effective oncological protocols.

## Graphical abstract

Over half of cancer patients undergo radiotherapy. Laser ablation enabled the synthesis of immiscible Au‐Fe‐B nanoparticles designed as degradable bimodal radiosensitizers for X‐ray radiotherapy (XRT), boron neutron capture therapy (BNCT), and bimodal imaging for X‐ray computed tomography (CT) and magnetic resonance imaging (MRI). These nanosensitizers respond to the problems of nanomaterials biopersistence, hypoxic tumors resistant to XRT, and imaging‐guided dose planning.

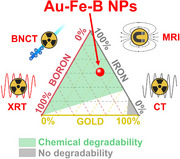

## 1 Introduction

Radiotherapy, used either alone or combined with other treatments, is a pivotal approach in modern oncology [1, 2]. In particular, X‐ray radiotherapy (XRT) is a key part of the clinical plan for more than 50% of all oncological patients, preferentially those affected by solid tumors or some haematologic malignancies like lymphomas [1, 2]. Yet, the efficacy of XRT is severely limited by its therapeutic window, which represents the interval between the minimum radiation dose for the effective destruction of cancer cells and the maximum dose tolerated by the surrounding healthy tissues [1, 2, 3, 4]. Additionally, XRT efficacy decreases in hypoxic tumors, which have low oxygen levels and low permeability to external drugs [3, 4, 5]. In fact, XRT requires the presence of molecular oxygen for the generation of reactive oxygen species (ROS), which can diffuse into the nuclei of cancer cells to induce irreversible damage to their DNA, mostly by a double strand break [3, 4]. The combination of a tight therapeutic window and hypoxic tumor regions multiplies the probability of recurrence, dramatically decreasing the chance of patient survival [1, 2, 5].

The accumulation of radiosensitizers in the tumor can expand the therapeutic window by locally amplifying the effect of X‐rays only where it is needed [3, 4]. Nanotechnology has offered innovative and advanced solutions for X‐ray radiosensitization, especially based on high‐Z biocompatible elements like Au [3, 6, 7, 8, 9, 10]. Nonetheless, the challenge of low oxygen concentration in the hypoxic tumor region remains unsolved [5]. To this end, alternative radiotherapy techniques, which are independent of the intratumoral oxygen concentration, are required to act synergistically with XRT. According to a large‐scale analysis on the application of inorganic nanoparticles in cancer therapy, multi‐therapy approaches are, in general, more effective than single‐therapy ones [10].

One type of radiotherapy, which is increasingly gaining attention for its efficacy in the treatment of normoxic and hypoxic tumors is boron neutron capture therapy (BNCT) [11, 12, 13, 14, 15]. BNCT is a binary hadrontherapy exploiting the high neutron capture cross section of the ^10^B isotope (19.9% natural abundance), to activate a nuclear reaction that releases an alpha particle and a lithium ion [11]. These charged particles have a high linear energy transfer (LET) and a traveling range comparable to the dimensions of the cells (ca 10 µm), as required to reach the cell nucleus where they induce double strand beaks in DNA and other types of complex chromosome aberrations [11, 16, 17]. Overall, this results in a greater biological effectiveness than X‐rays [16]. The application of BNCT has significantly accelerated in the last years due to the availability of clinical accelerators for the generation of epithermal neutrons [11, 12, 18]. However, a therapeutic window can be observed for BNCT only when the concentration of ^10^B in the tumor is at least 2–3 times higher than in normal tissues, which is possible by administration of boronated compounds as radiosensitizers [11]. Thus, the quantification of intratumoral ^10^B is necessary for treatment planning, but clinical boron compounds are not detectable with standard imaging techniques such as X‐ray computed tomography (CT) or magnetic resonance imaging (MRI) [11, 18, 19]. This forces to complex clinical protocols with delayed administration of radiolabeled boron carriers for positron‐emission tomography imaging [20], worsening the concerns about their biopersistence [21]. Besides, the anatomical precision in delivering the neutron beam to the tumor is lower compared to X‐rays, explaining the higher risks of recurrence despite the superior biological effectiveness [22, 23, 24, 25]. To date, only a few preliminary investigations focused on the advantages of combined BNCT and XRT to compensate for the dose heterogeneity (“cold spots”) associated with BNCT alone on tumors with large volume [22, 23, 24, 25], with preliminary indications of longer survival times in patients [25]. The range of benefits expected from the combination of XRT and BNCT is still far from being understood, for instance, including the increased tumor permeability upon exposure to XRT sensitizers [6], which may facilitate the diffusion of boron sensitizers in the hypoxic tumor region.

In this context, polymer‐coated Au‐B NPs have been recently proposed as nanotechnological radiosensitizers combining the advantages of XRT and BNCT [8]. The Au‐B NPs incorporated 2–3 orders of magnitude more boron than clinical BNCT sensitizers, while retaining also the other advantages of gold, such as the easy surface functionalization, indispensable to confer stability and molecular selectivity to the radiosensitizer, and enabling the CT tracking. The latter is crucial for providing a signal linearly correlated to the concentration of NPs, namely of the boron payload, in the tissues. However, CT is a low‐sensitivity technique that requires a relatively large concentration of contrast agent to avoid a loss of analytical reliability, especially for the quantification of radiosensitizers near dense tissues [26]. Besides, despite their immiscibility, B‐doped Au NPs are stable like pure gold and, therefore, introduce the problem of the long‐term persistence of inorganic nanomaterials in the body. NPs made only of Au are renowned for their useful properties and tolerability in the short term [27, 28, 29], but are not biodegradable, resisting corrosion even in the complex physiological environment of living organisms [30, 31, 32]. Indeed, it was previously demonstrated that Au‐Fe alloys are biodegradable in the appropriate conditions of composition (> 30 at% of iron) and atomic ordering (incomplete iron segregation preferable to homogeneous alloying), while also behaving as bimodal contrast agents for CT and MRI [31]. The molar sensitivity of MRI is up to 50 times larger than CT, in addition to being complementary to CT for the imaging of soft and dense tissues, respectively [26, 33]. Biodegradability is a property observed also in other Fe alloys, such as Fe‐B NPs, which were developed as BNCT radiosensitizers trackable by MRI [19]. The chemically degradable nature of these iron nanoalloys provided an ideal solution to the dilemma of nanomedicine [31, 34, 35], combining a size > 10 nm for the duration of theranostic function, with a continuous size reduction in the weeks following their administration to facilitate clearance without a massive and harmful accumulation in the kidneys. Biopersistence is one main issue of nanomedicine [10, 34, 35, 36], because cell uptake is more efficient for NPs above the 5–10 nm threshold [35, 37], but they also accumulate massively in the organs of the mononuclear phagocyte system (MPS) as the liver and spleen [10, 34, 35, 36], where they persist for indefinite time eliciting inflammation and ROS generation [34, 35, 37]. The persistence in the MPS organs is the first obstacle to the efficacy of nanomaterials because it prevents the accumulation in the tumor [10, 34]. On the other hand, NPs with a size below 5–10 nm have a relatively limited cellular uptake and undergo accelerated renal clearance or hepatobiliary excretion, with several risks connected to the fast accumulation in these organs, inflammation, and overall compromising renal functions [38, 39, 40]. Up to 70%–90% of the administered dose of renal clearable NPs pass through the kidneys in the first 24 h [39, 41], a stressful and risky process for these organs, which is not compatible with patients with compromised renal function, one typical side effect of chemotherapy and of the clinically used molecular contrast agents [38, 39, 42]. Polymer capsules with ultrasmall (2–3 nm) Au NPs have been proposed for delaying the clearance of ultrasmall Au‐based nanomedicines [43], but the encapsulation inside a dense matrix is not suitable for the radiosensitization function, which is maximized when the Auger electrons emitted from the NPs can interact with water molecules [3, 4].

Therefore, in this work, we address the challenge of combining all the advantages of the previously reported B alloys to obtain a bimodal nanosensitizer for XRT and BNCT, that is trackable and quantifiable by MRI and CT, and chemodegradable. The combination of three elements with completely different chemical and physical properties, such as Au, Fe, and B in a single NP is extremely challenging. Au is not thermodynamically miscible with Fe and B [44]. Although Au can form metastable alloys with both, these are, respectively, substitutional Au‐Fe alloys [31] and Au nanocrystals doped with interstitial or atomic‐scale segregated B [8], while Fe and B easily form amorphous and crystalline compounds [19, 45]. The completely different atomic ordering for each pair of elements adds to the different standard reduction potential and consequent reactivity with oxygen of Au, Fe, and B. The challenge of achieving all the desired functions in the Au‐Fe‐B NPs is further worsened by the simultaneous requirement of chemical degradability and exploitability. The chemical degradability requires that the trimetallic NPs possess a composition and atomic‐scale structure (also referred to as ultrastructure) favorable to corrosion in physiological media [31]. The exploitability imposes that the NPs must also resist for the time required for synthesis, functionalization, administration, and interaction with tumor cells [34, 36].

In this unfavorable scenario, the synthesis by pulsed laser ablation in liquid (PLAL) comes to the rescue. PLAL is considered a low‐cost, environmentally friendly technology for the synthesis of NPs at room temperature and pressure, without the need for chemical precursors [12, 46, 47, 48, 49]. In particular, PLAL has been established as a versatile technique for the realization of multielement nanocrystals and nanoalloys, including non‐equilibrium phases and high entropy alloys [47, 50, 51, 52]. This is possible because NPs formation in PLAL occurs on the timescale of only hundreds of µs, starting from the ablation of a bulk target immersed in a liquid environment [46, 47, 48]. The composition and physical‐chemical properties of the liquid are selected according to the specific reactivity of the elements in the final NPs [46, 47, 48, 53]. Since chemical precursors or stabilizers are not necessary, the functionalization of the NPs after or during the PLAL is easy, as required to confer stability to nanomedicines [8, 31, 54]. The trimetallic nanomedicine platform resulting from this approach represents a sophisticated and innovative tool for multimodal radiotherapy with X‐rays and neutrons, which also integrates the essential bimodal imaging abilities for real‐time tracking of the radiosensitizers inside the tumor. This nanomedicine has the required and long‐awaited features for leveraging new studies and advances in the precision and effectiveness of radiotherapy, ultimately providing a comprehensive solution for personalized oncology.

## 2 Results

### 2.1 Synthesis and Structural Characterization of Au‐Fe‐B NPs

**FIGURE 1:**
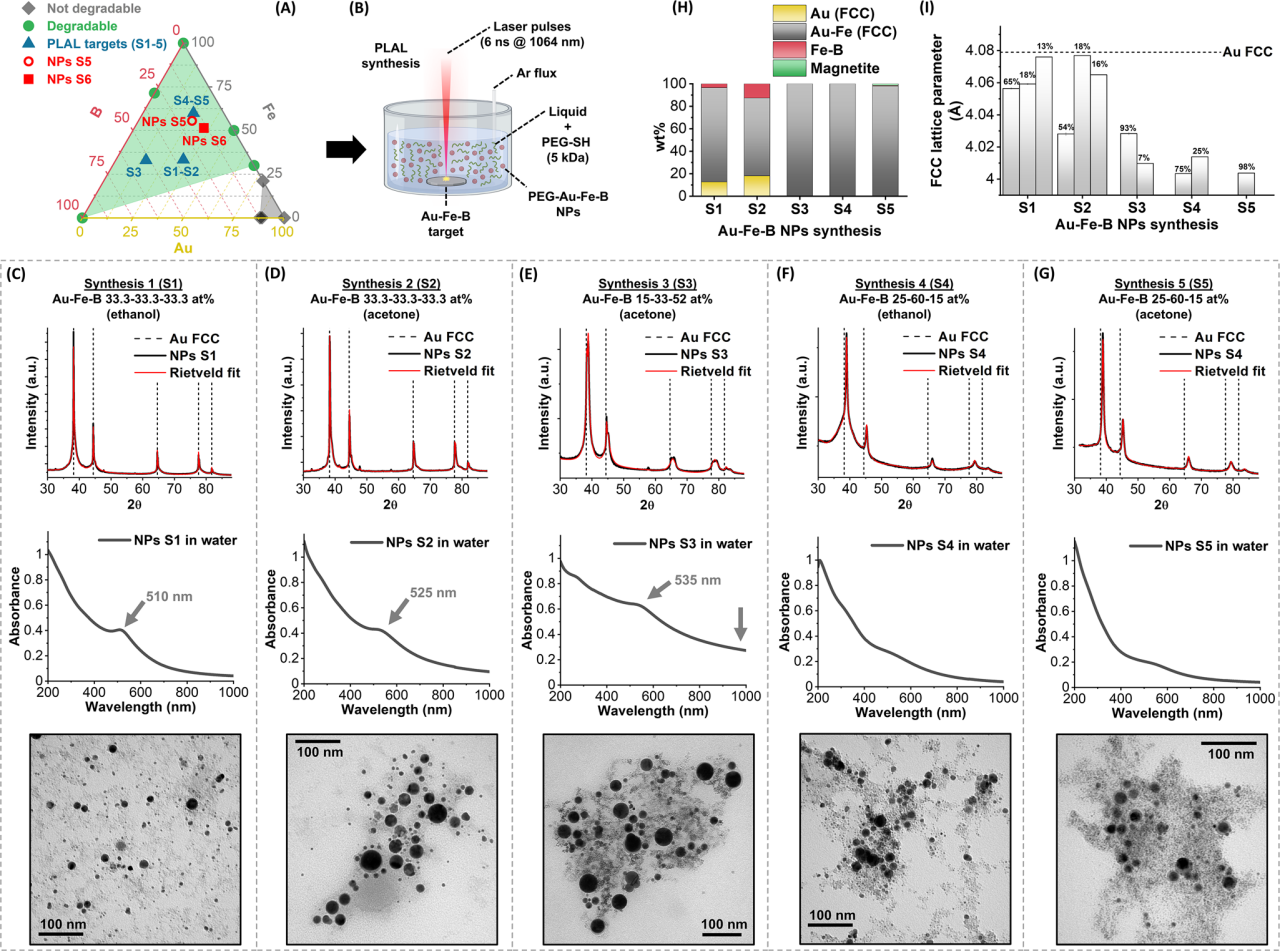
PLAL synthesis optimization. (A) Ternary diagram of the Au‐Fe‐B highlighting the regions where chemical degradability is (green) or is not (gray) expected. Green dots represent chemodegradable single or binary compounds according to literature [19, 31, 57, 58, 59, 60, 61]. Gray diamonds represent not chemodegradable single or binary compounds according to literature [8, 30, 31]. Blue triangles indicate the composition of bulk targets used for synthesis S1‐5. The composition of the NPs S5 and of their refinement (S6, described later) are indicated by red symbols. (B) Sketch of the PLAL synthesis. Created with biorender.com. (C–G) XRD (top), UV–vis (middle), and representative TEM image (bottom) of the NPs obtained with the five synthesis protocols S1‐5. The Rietveld refinement is indicated with the red curve in the XRD graphs. Arrows in UV–vis spectra indicate the LSP typical of Au alloys with marked Au character (C–E). The second arrow in (E) indicates the scattering background of this sample. (H) Phase composition according to the Rietveld refinement for the S1‐5 samples. (I) Comparison of the FCC lattice parameters obtained with the Rietveld analysis for the S1‐5 samples, indicating that in S5 just one component and with the highest deviation from the pure Au lattice parameter was obtained.

Departing from previous positive results about the synthesis of Au‐Fe [31], Au‐B [8], and Fe‐B [19] nanoalloys, the Au‐Fe‐B NPs were obtained by PLAL of a bulk trimetallic target dipped in an organic polar liquid (Figure 1). In PLAL, the NPs are formed by quenching the hot liquid metal droplets ejected from the bulk target during the ablation process, simultaneously with the condensation and coalescence of the ablated matter [55]. Therefore, the liquid environment should limit as much as possible the oxidation of Fe and B, while also allowing for the stability of the colloid. The colloidal state is one of the benefits of the PLAL approach compared to NPs agglomeration into a solid powder, typical of other non‐equilibrium physical synthetic methods successfully applied to polyelemental NPs [12, 56]. Previously, ethanol has been used for the PLAL synthesis of Au‐Fe and Au‐B nanoalloys [8, 31], whereas anhydrous acetone under Ar flux resulted in a more conservative environment for the Fe‐B nanosystems [19]. Nonetheless, the compositions of the binary nanoalloys often differed from that of the bulk target because of the oxidation of Fe and B. In a large part, this is due to the portion of the ablated matter that is released as vapors and ineluctably reacts with the surrounding solution species, such as trace oxygen and water molecules. To account for this phenomenon, the composition of the trimetallic Au‐Fe‐B target should include an excess of the more reactive components as Fe and B. Besides, the composition of the final Au‐Fe‐B NPs is critical for achieving chemical degradability, which occurred for Au‐Fe above 30 at% of Fe [31], and was observed also in Fe‐B 71‐29 at% [19] and iron oxide NPs [57, 58, 59]. Boron NPs also dissolve slowly into boric acid at slightly acidic pH and in the presence of oxidizing compounds, such as ROS generated within cells [60, 61]. Conversely, NPs of Au, Au‐Fe 79‐21 at%, and Au‐B 88‐12 at% have not shown chemical degradability [8, 30, 31]. Hence, the ternary Au‐Fe‐B diagram can be divided into a region of expected chemical degradability (green area in Figure 1A) and a region of expected structural stability (gray zone in Figure 1A).

The PLAL was performed in the batch configuration adopted for the other alloys [8, 19, 31] (Figure 1B), focusing near‐infrared ns laser pulses on trimetallic targets well within the range of expected chemical degradability (blue triangles in Figure 1A). Three targets with different composition were used: (i) equimolar in composition (S1 in ethanol and S2 in acetone with Au‐Fe‐B 33.3:33.3:33.3 at%, Figure 1C,D); (ii) with an excess of boron over iron and gold (S3 in acetone with Au‐Fe‐B 15:33:52 at%, Figure 1E); (iii) with an excess of iron over gold and boron (S4 in ethanol and S5 in acetone with Au‐Fe‐B 25:60:15 at%, Figure 1F,G). Thiolated polyethylene glycol (PEG‐SH) was dissolved in the liquid solution to react with the surface Au atoms and stabilize the NPs already during the PLAL, as well as when they are transferred into the aqueous solution. In fact, after PLAL the NPs were collected by centrifugation, washed to remove excess PEG (see Experimental Section for details), and analyzed by X‐ray diffraction (XRD) or resuspended in pure water for UV–vis spectroscopy.

The metallic nature of the NPs is indicated by the Rietveld refinement of the XRD pattern (Figure 1H,I), with a net prevalence of the face centered cubic (FCC) structure typical of pure Au and its alloys, although the reflections of crystalline FeB were also found in S1 (4 wt.%) and S2 (13 wt.%), and those of magnetite in S5 (2 wt.%). The contraction of the FCC lattice parameter (Figure 1I) is indicative of Au alloying with Fe, because the two elements can form a metastable substitutional solid solution. However, more than one FCC component was found in all samples except for the S5 (Figure 1I). The largest cell parameters were found in S1 and S2, where some components are equivalent to pure Au (Figure 1I). Intermediate cell parameters are measured in S3 and S4, and the smallest values are found in S5. Hence, the S5 resulted to be the sample with the best homogeneity and the largest amount of substitutional iron in the FCC lattice.

Additional analysis to confirm the XRD results was conducted by UV–vis spectroscopy, which is insightful for the assessment of Au nanoalloys because of the strong influence of the composition on the localized surface plasmon (LSP) absorption band [31, 62]. In samples S1, S2, and S3, a LSP band is observed in the 510–535 nm range, which is indicative of the presence of Au NPs with traces of dopants (Figure 1C–E). Instead, the plasmon band of samples S4 and S3 is broad and damped, as expected for Au solid solutions containing a consistent amount of Fe (Figure 1F,G). The near infrared (NIR) absorption in samples S4 and S5 decays to zero (Figure 1F,G), as expected for a well‐dispersed colloid, which is not the case, for instance, of the S3 sample, where large agglomerates induce a consistent scattering contribution in the NIR (Figure 1E).

**FIGURE 2:**
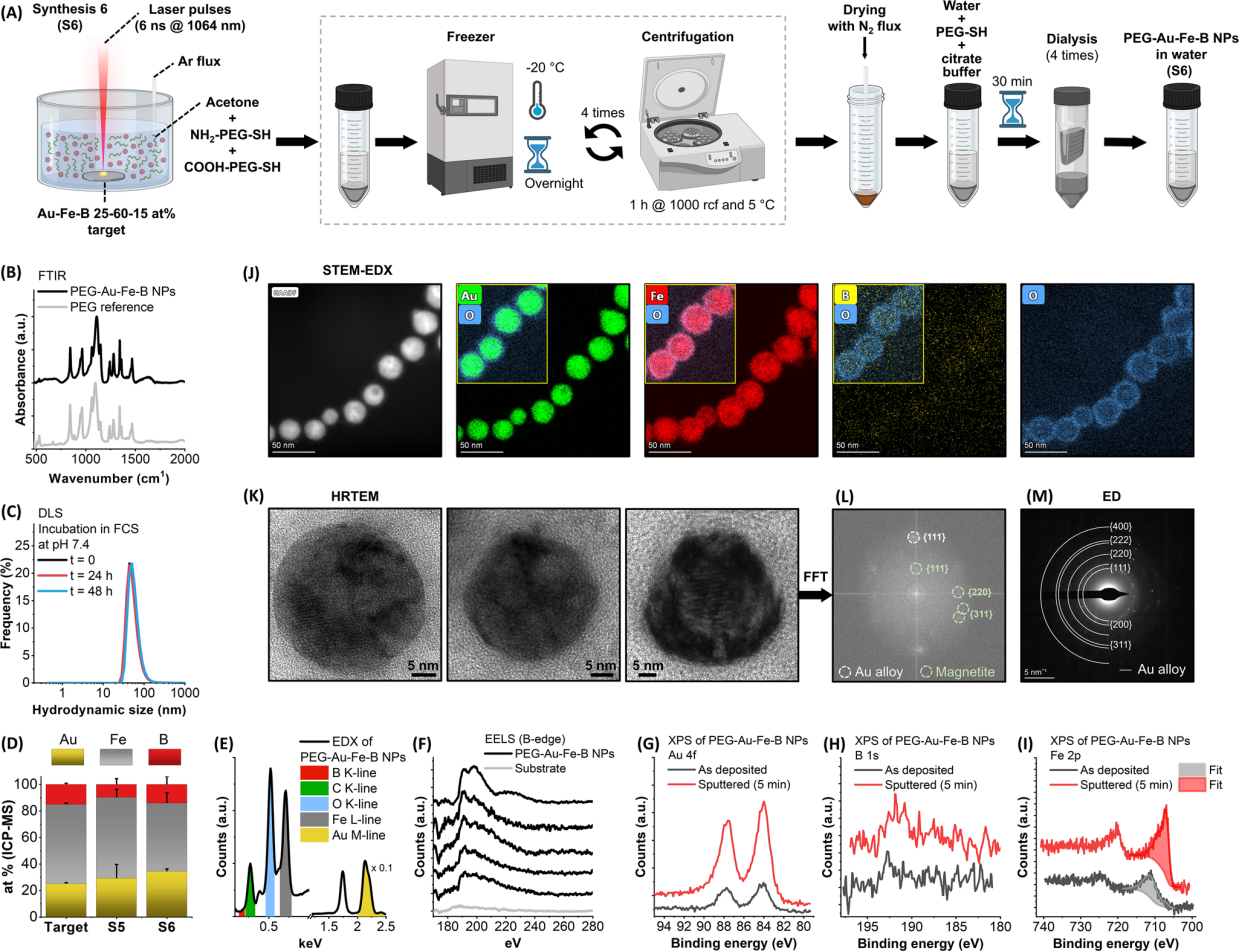
Optimized synthesis protocol and characterization. (A) Optimized synthesis protocol for PEG‐Au‐Fe‐B NPs S6. Created with biorender.com. (B) FTIR spectrum of the NPs, compared to that of the reference PEG coating. (C) DLS of the PEG‐Au‐Fe‐B NPs at 0, 24, and 48 h incubation in FCS. (D) ICP‐MS elemental analysis of the NPs S6 compared to the S5 sample and the original target. (E) EDX analysis of the NPs dried on a Si substrate. (F) EELS B‐edge measured on five randomly selected individual NPs. The sample grid background is also shown (gray line). (G–I) XPS analysis of Au 4f, B 1s, and Fe 2p peaks before (black lines) and after Ar^+^ sputtering (red lines). Collected with Al Kα source. The fitting of the Fe 2p_3/2_ peaks is shown as, respectively, gray and red areas. (J) STEM‐EDX net maps of the NPs, showing the HAADF image, the Au, Fe, B, and O signals. Insets show the overlap of O signal with the corresponding element. Note that the B EDX signal is intrinsically weak and results in low intensity compared to the net map background; hence, EELS analysis on individual NPs was also performed. (K) HRTEM images of the NPs and (J) FFT of the last NP, showing the reflections of FCC Au and magnetite. (M) ED pattern collected on a group of NPs, showing the reflections of the FCC phase.

Despite the positive indications about alloy formation in the NPs S5, all samples are composed of a mixture of spherical metal NPs and a network of low‐density compounds originating from the aforementioned oxidation of Fe and B (see transmission electron microscopy images, TEM, in Figure 1C,G). Chemical etching with chelating agents is usually used for cleaning corrosion‐resistant NPs from the low‐density network [54, 62]. Unfortunately, this is not possible for chemodegradable NPs like Au‐Fe nanoalloys, which have been previously purified through centrifugation [31], whereas Fe‐B nanoalloys were cleaned by incubation in a citrate buffer [19]. Here, only a combined approach succeeded in separating the Au‐Fe‐B NPs from their byproducts, without irreparably compromising the soft and chemically degradable nature of the nanoalloys (Figure 2A; Figure S1, see Experimental Section for details). The synthetic procedure, leading to the PEG‐Au‐Fe‐B NPs formulation S6, included a sequence of freezing, centrifugation, drying, incubation with citrate buffer at pH 4.7 in an aqueous environment for 30 min and, finally, repeated dialysis cycles until achieving the NPs in pure water. Besides, the PLAL protocol leading to the NPs S6 was modified compared to the NPs S5 using a mixture of bifunctional PEGs (NH_2_‐PEG‐SH and COOH‐PEG‐SH) instead of the PEG‐SH, to endow the polymeric shell of the NPs with a surface charge density at both acidic and basic pH. This is aimed at improving the colloidal stability of the NPs in the various physiologic environments relevant to cancer theranostic applications [5].

The Fourier transformed infrared (FTIR) spectrum of the sample S6 contains the same vibrational fingerprint of the pure polymers [8, 31], confirming the successful coating of the NPs with the PEG derivatives (Figure 2B). Dynamic light scattering (DLS) of the NPs S6 indicated a nanometric hydrodynamic size comprised between 30 and 100 nm, minimally affected by the incubation with foetal calf serum (FCS) for 24 or 48 h (Figure 2C). The elemental composition of the NPs S6 was measured with inductively coupled plasma‐assisted mass spectroscopy (ICP‐MS, Figure 2D), resulting in Au 34 ± 2 at%, Fe 52 ± 8 at%, and B 14 ± 6 at%. As indicated by the ternary Au‐Fe‐B diagram in Figure 1A, the NPs S6 remain well within the region of expected degradability and have only minor composition differences with the NPs S5 and the target. The composition of S6 is close to that of S5 (Au 29 ± 10 at%, Fe 61 ± 6 at%, and B 10 ± 4 at%), except for a reduction in the iron content, suggesting that the low‐density network observed in TEM images of Figure 1G was rich in Fe. The elemental composition of the inorganic core (Au M‐line, Fe L‐line, B K‐line) and of the organic surface coating (C K‐line and O K‐line) of NPs S6 is also confirmed by energy dispersive X‐ray spectroscopy (EDX) on the sample dried at room temperature on a monocrystalline Si substrate (Figure 2E). Since the EDX signal of boron has low intensity and overlaps with the tail of the C peak [8, 19], its presence was also confirmed at the single NPs level by electron energy loss spectroscopy (EELS, Figure 2F) [8, 49].

The chemical nature of Au, Fe, and B was identified with X‐ray photoelectron spectroscopy (XPS) with an Al Kα source, both on the as‐deposited sample and after 5 min of sputtering with Ar^+^ ions (XPS, Figure 2G–I). The Au 4f line is proper of gold in its metallic state and remained unchanged before or after sputtering (Figure 2G) [8]. Boron has an intrinsically weak photoemission intensity and, in the as‐prepared sample, its 1s line is broad and centered at a binding energy (BE) of approximately 192.5 eV, which corresponds to B_2_O_3_ (Figure 2H) [8, 63]. The formation of an amorphous surface layer of boron oxide has been systematically observed in metal borides [45]. After sputtering, the B 1s peak is still centered at the BE of boron oxide, although with a broadening to lower energies, indicating a possible component with a reduced state reminiscent of what previously found in Au‐B NPs [8, 63]. Importantly, the signal‐to‐noise ratio of the B 1s photoemission line increased after sputtering away the polymeric coating and the very first nanometers (3–4 nm) of NPs surface, indicating that boron is located throughout the NPs and not just at their surface. The surface oxidation of the NPs is further confirmed by the analysis of the Fe 2p line, which has an iron oxide character in the as‐deposited sample, whereas a pronounced metallic feature at 707 eV appears after sputtering, as expected for an Au‐Fe alloy [64]. The iron photoemission corresponds to 93 at% of magnetite with an excess of Fe^3+^ and 7 at% of metallic iron in the as‐prepared sample. After sputtering, the share of Fe phases becomes 53 at% of magnetite with an excess of Fe^2+^ and 47 at% of metallic iron. The presence of a metallic Au‐Fe alloy phase embedded in a shell of iron and boron oxides is confirmed by the B/Au atomic ratio (0.6 for the as‐prepared sample and 0.23 after sputtering) and the Fe/Au atomic ratio (1.1 for the as‐prepared sample and 0.6 after sputtering). However, in the case of samples constituted by a deposit of polymer‐coated NPs, the surface stoichiometry can only be indicative of a qualitative trend. In consideration of this issue, and of the intrinsically low signal of B 1s, the XPS measurements were repeated with another X‐ray source (Mg Kα, see Figure S2), which fully confirmed the boron oxidation state, its signal increase after sputtering, and the trend of the B/Au ratio before and after sputtering.

The scanning TEM EDX maps (STEM‐EDX, Figure 2J) and high‐resolution TEM (HRTEM, Figure 2K) confirm at the single NP level the structural insights obtained from XPS. In particular, a shell of Fe and O is clearly detected around a core containing Au, Fe, B, and O (Figure 2J). The HRTEM images of NPs (Figure 2K) reveal that the metallic cores are not homogeneous because they contain several less electron‐dense regions. This morphology was previously observed in the initial steps of Au‐Fe NPs chemical degradation [31], and it is expected due to the 30 min incubation of the Au‐Fe‐B NPs in citrate buffer. According to the STEM‐EDX maps, the O K line is more intense in the shell of the NPs but is also found inside them, indicating that the Au‐Fe metallic scaffold is embedded in the Fe and B oxide phases. The fast Fourier transform (FFT) of the HRTEM image allows identifying the reflections of the FCC Au alloy and iron oxide (magnetite), confirming further that the two phases are contained within the same NPs (Figure 2L). The electron diffraction (ED) pattern collected on an ensemble of NPs, however, is dominated by the FCC Au alloy reflections (Figure 2M).

### 2.2 Chemodegradable Nature and Functional Properties of Au‐Fe‐B NPs

**FIGURE 3:**
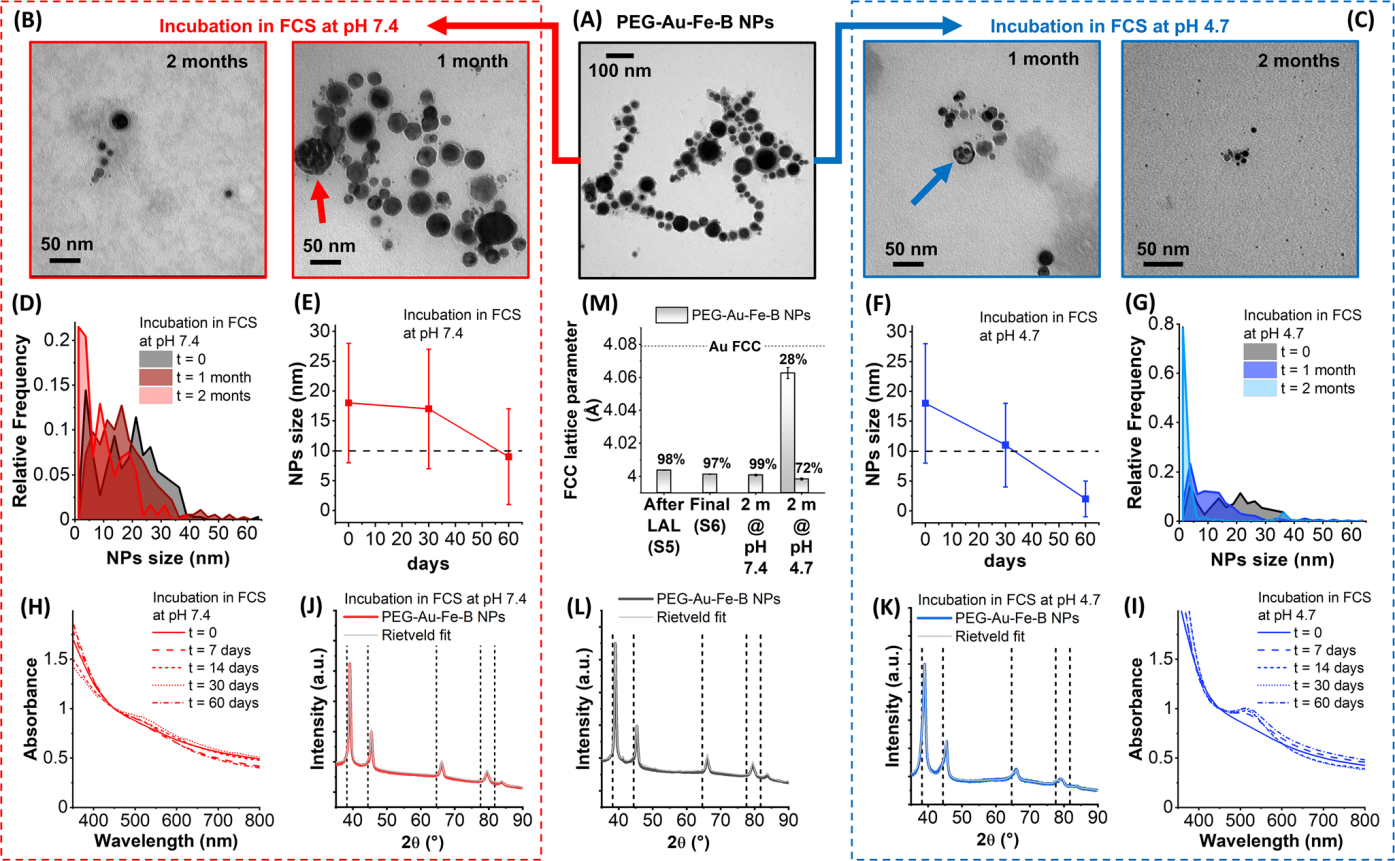
Structural evolution in physiological environments. (A–C) Representative TEM image of the PEG‐Au‐Fe‐B NPs sample before (A) and after incubation in FCS at pH 7.4 (B) and 4.7 (C) for 1–2 months at 37°C. (D–G) Size distribution and average size with relative standard deviation of the NPs at 0, 1, and 2 months of incubation at pH 7.4 (D,E) and 4.7 (F,G). (H,I) UV–vis at the different incubation timepoints at the two pH. The spectra were normalized at 450 nm for ease of comparison. (J–L) XRD with the corresponding Rietveld fit (gray lines) of the Au‐Fe‐B NPs after 2 months of incubation at pH 7.4 (J) and 4.7 (K), and at time 0 (L). (M) The FCC lattice parameters obtained from the Rietveld refinement of XRD patterns of NPs S6 at time 0 and after 2 months incubation at the two pH (2m@pH7.4: incubated for 2 months at pH 7.4; 2m@pH4.7: incubated for 2 months at pH 4.7; labels indicate the corresponding wt.%). The lattice parameter of the NPs S5 is also shown for comparison.

The chemically degradable nature of the Au‐Fe‐B NPs, already suggested by the structural characterization, was specifically investigated by UV–vis spectroscopy, XRD, and TEM after aging the S6 sample in FCS at physiological (7.4) and lysosomal (4.7) pH at 37°C (Figure 3) [19, 31, 43, 54, 59]. Slightly acidic conditions (up to a pH 5.5) and high ROS concentration also occur in the tumor microenvironment of several types of cancer. In most cases, the pH of the tumor microenvironment is 6.5 [5]. Therefore, the ageing of the S6 sample in FCS at pH 6.5 and 37°C was also tested (Figure S3). The protocol for assessing the structural evolution of NPs under conditions mimicking the biological environment is the same as previously reported in literature [59, 65, 66], and showed its reliability in predicting biodegradability in vivo in three other types of alloy nanoparticles (Au‐Fe [31], Ag‐Fe [54], Fe‐B [19]).

The TEM images collected after 1 month show that several NPs underwent corrosion and exhibit pores or crescents due to the incomplete dissolution of the original spherical morphology (Figure 3A–C; Figure S3). Besides, the pristine oxide shell is observed only around some of the NPs at physiological pH but not at lysosomal pH, where a significant size reduction is measured. After 2 months, the size of the NPs decreased from the initial value of 18 ± 10 nm to 9 ± 8 nm at pH 7.4 and 2 ± 3 nm at pH 4.7, which is below the threshold for renal clearance (Figure 3D–G) [36, 38, 39, 40]. In this case, the fraction of ultrasmall particles (< 5 nm), which is < 16% in the S6 sample, after 2 months of incubation becomes 42% at pH 7.4, 72% at pH 6.5, and 95% at pH 4.7 (Table S1). The NPs with size < 5 nm are more easily excreted via kidneys, whereas those > 5 nm are preserved for months and even years in liver, spleen, and other tissues, where they are phagocytized by phagocytes and Kupfer cells [29, 32].

It should be noted that the TEM images of the S5 sample also revealed the presence of numerous ultrasmall particles alongside the primary Au‐Fe‐B NPs (Figure 1G). The EDX analysis indicates that the ultrasmall particles in S5 have a similar composition of primary Au‐Fe‐B NPs (Figure S4), explaining why they were chemically degraded by the acidic treatment in the synthetic protocol leading to the S6 sample, except for a residual fraction found only around the primary larger NPs (Figure 3A). This is not the case of the NPs < 5 nm observed in the S6 sample incubated for two months; in fact, the UV–vis and XRD analysis (Figure 3H–M) show that the morphological evolution takes place also by the release of substitutional iron from the FCC Au alloy. For instance, their LSP band becomes more evident after ageing in the two environments (Figure 3H,I), which is the opposite of the behavior expected from NPs undergoing size decrease. Hence, the appearance of an LSP can be explained only with the enrichment of the Au fraction in the FCC lattice [31, 62, 64]. The Rietveld refinement of the XRD patterns (Figure 3J–M) indicates that the pristine NPs S6 have the same FCC lattice parameter as the sample S5, with only a minor increment of the iron oxide component (from 2 to 3 wt.%). The NPs S6 aged at pH 7.4 did not show a change in the lattice parameter but only a decrease of the iron oxide phase to 1 wt.%. In the NPs aged at pH 4.7, instead, a second FCC component with a lattice parameter near that of pure Au appeared, as expected from the LSP in the UV–vis spectra.

Overall, despite the chemical degradation kinetics of the Au‐Fe‐B NPs required several weeks, this is an improvement compared to benchmark Au or iron oxide NPs, which have not shown any degradability on the same timescale in previous studies [19, 31, 54], motivating the interest in the development of this type of innovative metastable nanoalloys.

**FIGURE 4:**
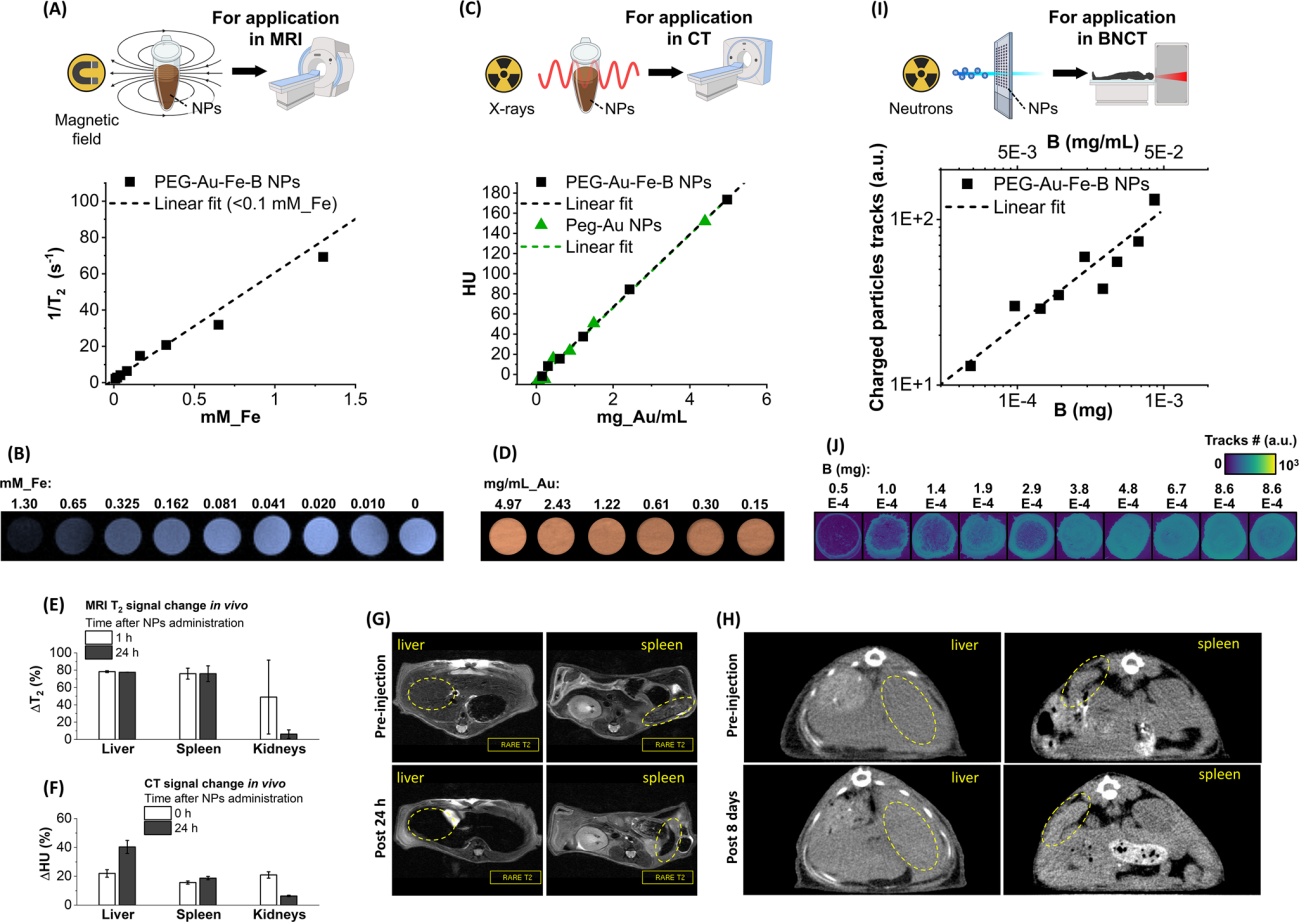
Functional assessment for application in MRI, CT, and BNCT. (A,B) Plot of transversal relaxation rate (1/T_2_) vs. Fe concentration collected on phantoms containing the PEG‐Au‐Fe‐B NPs sample at variable dilution (A) and MRI images of phantoms cross‐section (B). The dashed line in (A) represents the fit in the linear range at low concentration (< 0.1 mm of Fe). Sketches in (A,B) were created with biorender.com. (C,D) Plot of HU vs. Au concentration collected on phantoms containing the PEG‐Au‐Fe‐B NPs at variable dilution (C) and CT images of phantoms cross‐sections (D). The reference PEG‐Au NPs curve is also reported in (C). The linear fit is indicated by the dashed lines (black for Au‐Fe‐B and green for Au) in (C). (E) Plot of the MRI contrast in vivo, measured as the relative T_2_ signal intensity decrease (ΔT_2_, expressed in absolute % variation), in the liver, spleen, and kidneys 1 h and 24 h after NPs administration. Error bars represent the SD. (F) Plot of the CT contrast in vivo, measured as the relative HU signal increase (ΔHU, expressed in absolute % variation), in the liver, spleen, and kidneys 0 h and 24 h after NPs administration. Error bars represent the SD. (G,H) MRI (G) and CT (H) images showing the change in contrast of liver and spleen after NPs administration. (I,J) Correlation between charged particle tracks generated upon epithermal neutron irradiation and the mass of PEG‐Au‐Fe‐B NPs deposited on CR39 films (I) and qualitative bidimensional map of charged particle tracks intensity (J). The dashed line represents the linear fit on the log‐log scale.

After the requisite of chemical degradability, the performances of the Au‐Fe‐B NPs for multimodal imaging and interaction with ionizing radiation were evaluated. The presence of iron in magnetic phases permits the use of the NPs as contrast agents for MRI due to the shortening of the transversal relaxation time (T_2_) of protons in the surrounding water molecules (Figure 4A,B) [19, 26, 31, 33]. In the linearity range at concentrations below 0.1 mm_Fe, the relaxivity resulted in 59 ± 1 s^−1^ mm_Fe^−1^, in line with the performance of other alloys based on Au and Fe [31]. The presence of Au confers to the NPs the ability to absorb X‐rays, generating a positive contrast in CT imaging (Figure 4C,D) [8, 19, 26, 27, 28, 33]. The CT signal, measured in Hounsfield units (HU), is linearly correlated to NPs concentration, with a slope of 36 ± 1 HU mL/mg_Au (as a comparison, the clinically used contrast agent based on iodine, iopromide, has a slope of 15.9 HU mL/mg_I) [67]. The bimodal MRI‐CT imaging ability was then tested in vivo in healthy mice injected intravenously with an NPs dispersion in PBS (0.2 mL at 150 mg/kg body weight). Both a T_2_ signal intensity decrease (ΔT_2_, expressed in absolute % variation, Figure 4E) and a CT signal increase (ΔHU, expressed in absolute % variation, Figure 4F) were measured in the liver, spleen, and kidneys soon after NPs administration (at 1 h for MRI and 0 h for CT). This change is indicative of particle distribution in the bloodstream. At 24 h after administration, the MRI and CT contrast remain stable in the liver and spleen, while decreasing in the kidneys. The NPs localization was monitored by CT until 14 days (Figure S5), indicating that the maximum accumulation in liver and spleen occurred 8 days after administration. Consequently, the T_2_‐weighted MRI images show that the liver and spleen are darker 24 h after NPs injection (Figure 4G), while the same organs reached the brightest contrast in CT images 8 days after injection (Figure 4H). The maximum of CT signal at 8 days and the decrease after 14 days agrees with the slow chemical degradation kinetics of the Au‐Fe‐B NPs described in Figure 3 and Figure S3.

The neutron capture (NC) ability of the Au‐Fe‐B NPs due to the presence of boron, and the consequent generation of charged particles upon interaction with a neutron beam, was measured through neutron autoradiography (Figure 4I,J, see Experimental Section for details). As expected, the number of charged particle tracks generated by ^10^B atoms in the sample scales with the mass of B in the deposited NPs (Figure 4I), simultaneously leading to an increment of the density map generated by the high‐LET particles on the radiosensitive substrate (Figure 4I,J).

### 2.3 In Vitro Assessment of Au‐Fe‐B NPs Efficacy

**FIGURE 5:**
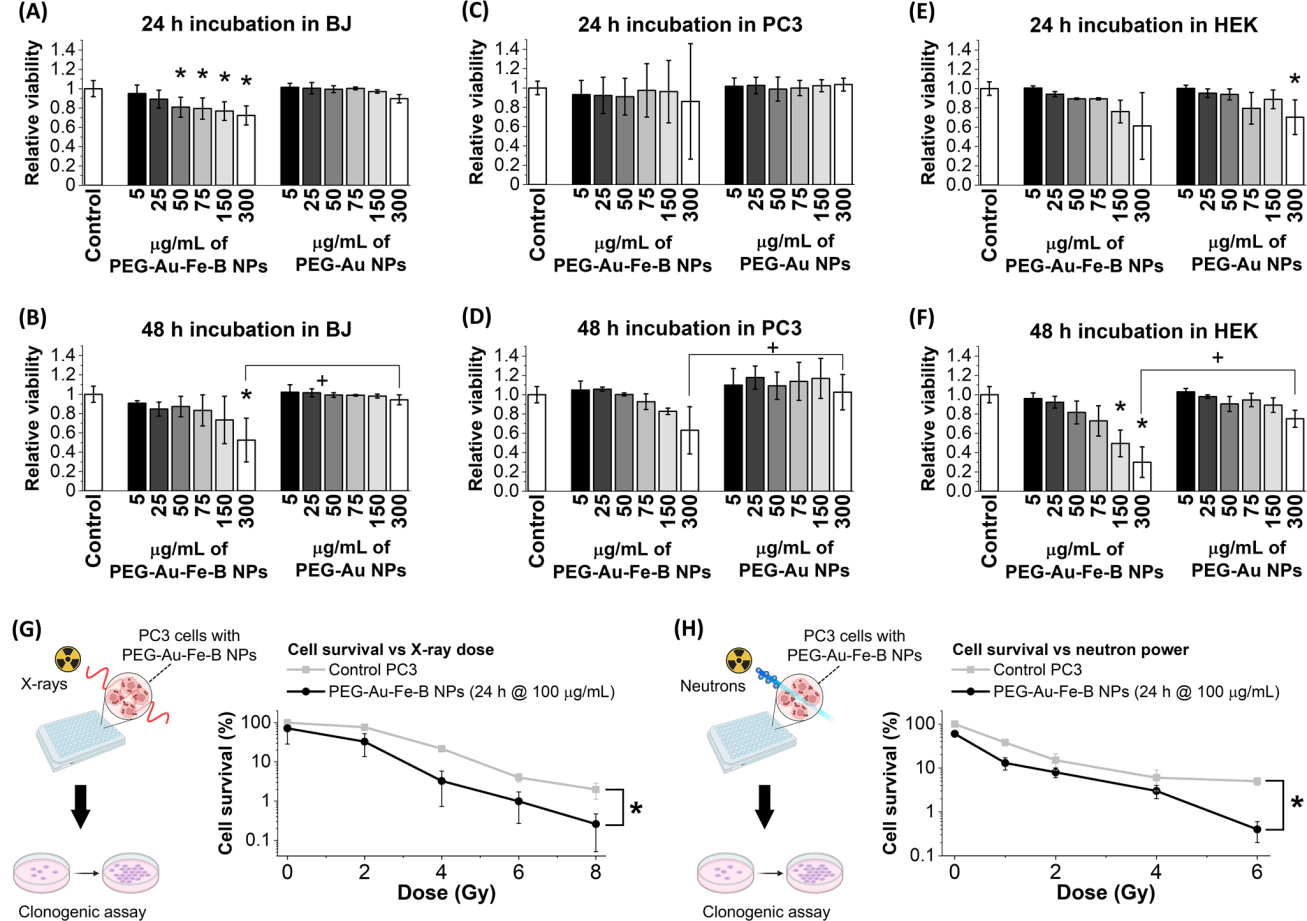
Cytocompatibility and radiotherapy assessment. (A–F) Cytocompatibility of PEG‐Au‐Fe‐B and reference PEG‐Au NPs incubated for 24 and 48 h with BJ (A,B), PC3 (C,D), and HEK (E,F) cells at various concentrations from 5 up to 300 µg/mL. Determined by MTT (N = 3, * represents *p* < 0.05 compared to the control, and + represents *p* < 0.05 compared to the same concentration of Au‐Fe‐B). The boxplots, the standard deviation (SD) – standard error (SE) comparison, and individual elemental concentration of Au, Fe, and B are reported in Figures S6 and S7. (G,H) Cell survival after in vitro XRT (G, N = 4) and BNCT (H, N = 3) experiments on PC3 cells incubated for 24 h with 100 µg/mL of PEG‐Au‐Fe‐B NPs. * represents *p* < 0.05 compared to the given pair of data indicated. Sketches in (G,H) were created with biorender.com.

After the positive outcome of the structural and functional assessment, the PEG‐Au‐Fe‐B NPs S6 were tested for cytocompatibility and bimodal radiotherapy in vitro. For cytocompatibility, normal (fibroblasts, BJ) and cancer (prostate carcinoma, PC3) cells were selected as representatives of, respectively, healthy and tumor tissues, and the effect of Au‐Fe‐B NPs was benchmarked against PEG‐coated Au NPs. Besides, human embryonic kidney (HEK) cells were selected as non‐tumoral cells with a proliferation rate comparable to that of tumor cells, because of their higher sensitivity to exogenous substances. While the PEG‐Au NPs have a complete cytocompatibility with BJ and PC3 cells in all the conditions tested, the PEG‐Au‐Fe‐B NPs were also well tolerated up to 150 µg/mL (vitality well above 70%, Figure 5A–D; Figures S6 and S7). The threshold of 150 µg/mL is more than one order of magnitude larger than in other Au‐based nanomedicines undergoing chemical transformation over time [68, 69]. Although 150 µg/mL is located at the very beginning of the dose‐response contrast curve of CT imaging (Figure 4C), it equals 0.79 mm_Fe, which is well within the range for an effective contrast in MRI (Figure 4A). This highlights the importance of having the bimodal contrast capability in the Au‐Fe‐B NPs for enabling their tracking, localization, and biodistribution in the broadest range of concentration possible. The most appropriate imaging technique can be selected depending on the sensitivity limits and the tissues under investigation [26, 70, 71]. For instance, CT can be used when NPs concentration is above the sensitivity threshold of 100–150 µg/mL, as shown in Figure 4C, whereas MRI provides information on the accumulation and biodistribution of the Au‐Fe‐B NPs also at a lower concentration.

The tolerability of HEK cells to the Au‐Fe‐B NPs was lower than that of fibroblasts and PC3, at 24 h (Figure 5E), and at 48 h only 5 µg/mL of Au‐Fe‐B NPs was tolerated (Figure 5F). Indeed, the polymer‐coated Au NPs also affected the vitality of the embryonic cells at the highest concentrations tested, which may be indicative of an effect related to the different uptake of the two types of NPs, as shown below, instead of the different chemical composition.

Hence, the potential of the S6 sample for radiosensitization in both XRT and BNCT was investigated using PC3 cells incubated for 24 h with the NPs (Figure 5G,H; Figures S8 and S9). The concentration (100 µg/mL) was selected since it was still in the range where no significant cytotoxicity is expected. The results of the clonogenic assays on the irradiated cells confirmed the efficacy in the enhancement of both the treatments with X‐rays and neutrons, because the viability of cells incubated with the NPs is systematically below the controls at all the radiation doses considered. The radiotherapy enhancement effect is due to the different elemental constituents of the Au‐Fe‐B NPs, namely Au atoms for XRT and B atoms for BNCT. While the radiosensitization mechanism of B has been described in the introduction, the radiosensitization with Au atoms exploits their large atomic number (Z = 79) and consequently large X‐ray linear attenuation coefficient [4]. Au atoms, after absorption of X‐rays, emit various types of ionizing radiation (photoelectrons, Compton electrons, Auger electrons) which can damage DNA directly or, with higher probability, indirectly by generation of ROS due to the ionization of water and oxygen molecules [4, 6].

**FIGURE 6:**
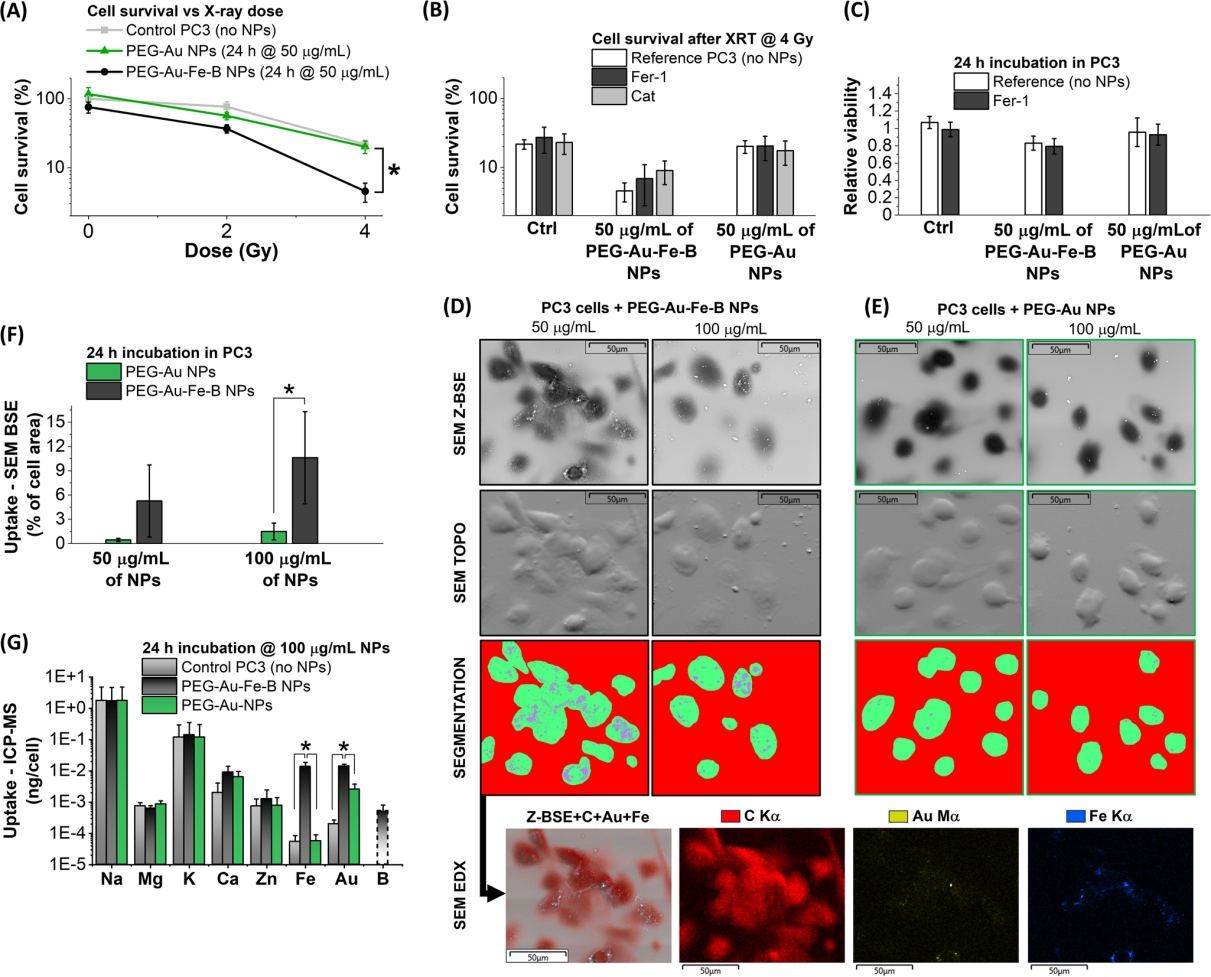
Cell interaction and uptake. (A) PC3 cell survival vs. X‐ray dose after incubation for 24 h at 50 µg/mL of PEG‐Au‐Fe‐B or PEG‐Au NPs. N = 4. * represents *p* < 0.05 compared to the given pair of data indicated. (B) Comparison of the PC3 cell survival for X‐ray irradiation at 4 Gy after incubation in the same conditions as (A) and in the presence of Fer‐1 or Cat. N = 4. No statistically significant differences were observed. (C) Comparison of PC3 cell viability after incubation in the same conditions as (A) and in the presence of Fer‐1. N = 4. No statistically significant differences were observed with *p* < 0.05 compared to, respectively, the control and the given pair of data indicated. (D,E) Representative SEM images of PC3 cells incubated for 24 h at 50 or 100 µg/mL of PEG‐Au‐Fe‐B (D) or PEG‐Au NPs (E), collected in Z‐BSE (maximization of Z‐contrast) and TOPO (surface topographic information) mode. The corresponding segmented images (green: cells; magenta: NPs; red: background region excluded from analysis) are also shown. A representative SEM‐EDX map of the PC3 cells incubated for 24 h at 50 µg/mL of PEG‐Au‐Fe‐B, showing the overlap of the C Kα signal primarily due to the cells and of Au Mα and Fe Kα signals due to NPs. (F) Evaluation of NPs uptake in the samples of (D,E), resulting from the average over a minimum of four segmented SEM images per each condition and expressed as the ratio of NPs surface over total cell surface. Error bars denote standard deviation. (G) Quantification of NPs uptake in PC3 cells incubated for 24 h at 100 µg/mL NPs by ICP‐MS measurement of Au and Fe content, in comparison with intrinsic elements such as Na, Mg, K, Ca, and Zn. B content in the cells treated with the Au‐Fe‐B NPs is obtained from Au and Fe values, considering the composition previously estimated with ICP‐MS on a pure NPs sample. N = 3.

The interaction of the Au‐Fe‐B NPs with the PC3 cells and the origin of their radiosensitization effects were investigated further with a series of experiments aimed at discriminating between the role of nonspecific physical and chemical effects related to NPs adsorption and internalization, and more specific biochemical effects such as ferroptosis, which are worth of consideration for iron‐based nanomaterials and nanoalloys [5, 35, 68, 72, 73]. To this end, the in vitro XRT treatment was repeated using the PEG‐Au NPs, since these are considered a benchmark for X‐ray radiosensitization [3, 4]. The nanomaterials were incubated with cells at 50 µg/mL to reduce possible nonspecific effects contributed by the exogenous NPs, as suggested by the cytocompatibility experiments. The XRT experiment showed a remarkable difference between the radiosensitization effect of the Au‐Fe‐B NPs and the absence of effects in the Au NPs treated cells, whose curve overlaps with the control (Figure 6A). Then, the irradiation was performed in the presence of a ferroptosis inhibitor (ferrostatin‐1, Fer‐1) and of a scavenger for H_2_O_2_ (catalase, CAT), because H_2_O_2_ is required in the Fenton reaction for the step of oxidation of Fe(II) to Fe(III) with the formation of more reactive ROS (hydroxyl radical and hydroxide ion) [5, 68, 69, 73]. However, no statistically significant changes were observed when cells were treated with Fer‐1 or CAT and X‐rays at 4 Gy, either in the control or the samples with Au‐Fe‐B or Au NPs (Figure 6B; Figure S10). Similarly, no statistically significant changes were observed in the survival rate of cells incubated with Fer‐1 and the NPs (Figure 6C), and the increase of vitality upon incubation with CAT and the Au‐Fe‐B NPs was only 4 ± 3%. This finding aligns with the results of the test of the intracellular level of hydrogen peroxide, a common ROS present in cells, after incubation for 24 h with Au‐Fe‐B NPs, showing no statistically significant changes with the control, both in PC3 cells not treated with X‐rays and those treated at 2 and 4 Gy (Figure S11).

Glutathione depletion is another common phenomenon at the origin of several processes leading to cell toxicity [72]. Glutathione is present in cells in both reduced (GSH) and oxidized (GSSG) forms, and its ratio is an indicator of oxidative stress. In this case, the GSH depletion was assessed in cells incubated for 24 h with the Au‐Fe‐B NPs at 50 µg/mL, indicating that the NPs altered the GSH/GSSG ratio, with a decrease to 20 ± 16% of the value in control cells not incubated with NPs (Figure S12).

According to the chemical degradation experiments reported in Figure 3, the release of iron from the NPs is slow compared to the timescale of cellular processes, thus explaining the lack of evidence for ferroptosis, which requires the presence of active Fe^2+^ [68, 72, 74]. Instead, GSH depletion is involved in several mechanisms leading to cell stress and death, for instance, GSH exhaustion can provoke mitochondrial oxidative stress and activation of the caspase cascade [75], which agrees with the reduction of mitochondrial metabolic activity evidenced by the cytotoxicity assay in Figure 5A–F. GSH depletion also acts as a generic factor leading to the amplification of other sources of cell stress, and, in this case, the Au‐Fe‐B NPs are subject to consistent cell uptake, thus introducing a source of interference with the normal cellular processes and organelle functions called “mechanical stress” [37, 72, 75, 76]. The combination of such multiple interpenetrated phenomena contributes to disrupting cell homeostasis and leads to the observed cytotoxicity, as was frequently reported about the interaction of cells with nanotheranostic agents under a phenomenon called panoptosis [72].

The different behavior of the two types of NPs was investigated further by the inspection of NPs uptake at the single cell level with environmental SEM (ESEM, Figure 6D,E), which does not require sample metallization or alteration. According to this analysis, the Au‐Fe‐B NPs (Figure 6D) undergo a consistent uptake by the PC3 cells, both at 50 and 100 µg/mL, as it is shown by the bright spots overlapped with the dark areas corresponding to cells in the backscattered electrons image (Z‐BSE image). In some cases, the perinuclear distribution of the NPs is also evident. The topographic BSE image of the same region (TOPO images) indicates that most of the Au‐Fe‐B NPs are inside cells (all those not detected in the TOPO image), whereas other NPs are well identifiable because located in proximity to the cell membrane. Besides, the EDX mapping confirms that the NPs S6 inside the cells are composed of both gold and iron, including those located in the perinuclear region. Unfortunately, the intrinsically low boron EDX signal could not be detected above the carbon background in this experiment. However, the presence of boron at the single‐cell level was confirmed by in vitro neutron autoradiography (Figure S13). Clusters of charged particle tracks generated by ^10^B atoms upon interaction with the neutron beam are observed in PC3 cells incubated for 24 h with the Au‐Fe‐B NPs at 50 µg/mL. The localization of the tracks along the cell is equivalent to that of the NPs, as assessed by the SEM analysis. Besides, no clusters of tracks are observed in the control PC3 cells, for which a significantly lower density of charged particles remained impressed on the radiosensitive CR39 substrate.

In the cells treated with the reference Au NPs (Figure 6E), a much lower uptake was detected, explaining the absence of a radiosensitizing effect. As shown in Figure 6D,E, an algorithm for image segmentation based on a supervised machine learning model (see Experimental Section for details) was applied to the SEM Z‐BSE images with the purpose of better evidencing the overlap between NPs and cell area. This method does not discriminate among NPs inside cells or on their membrane, but, on the other hand, it allows quantifying the fractional area occupied by NPs over the total cell area (Figure 6F). The results confirm that Au‐Fe‐B NPs interact more effectively with PC3 cells than the pure Au equivalents. This enhanced interaction is likely due to the chemodegradable nature of the nanoalloys, which has the potential to facilitate the loss of the stealth polymeric coating over time, permitting the cellular uptake, which did not occur with inert and non‐degradable sensitizers as the PEG‐Au formulation. In addition, the uptake of the NPs was measured with ICP‐MS (Figure 6F), indicating that the cell cultures treated with the Au‐Fe‐B NPs at 100 µg/mL contained 0.014 ± 0.002 ng/cell of Au compared to the 0.0025 ± 0.0012 ng/cell of those treated with Au NPs (in untreated cells, the background was at 2.0 ± 0.7 10^−4^ ng/cell). Iron was detected in the sample treated with Au‐Fe‐B NPs at a concentration of 0.014 ± 0.005 ng/cell, compared to 5 ± 3 10^−5^ ng/cell in the untreated cells (6 ± 3 10^−5^ ng/cell in the cells treated with Au NPs). Boron could not be directly measured by ICP‐MS because its inherently low signal‐to‐noise was decreased further by the dilution required for proper digestion of the cells [8, 19]. Despite this problem representative of the difficulties in direct boron dosage in cells and tissues, the B content associated with the Au‐Fe‐B nanosensitizers was evaluated indirectly but easily from the Au and Fe signals, resulting in 5 ± 3 10^−4^ ng/cell. This estimation does not discriminate between intracellular NPs and NPs adsorbed on the cell membrane, but it is noteworthy that 5 ± 3 10^−4^ ng/cell of boron corresponds to 10^10^ molecules of boronophenylalanine (BPA) or 10^9^ molecules of borocaptate, the two sensitizers used in clinical BNCT [11, 20].

Finally, the X‐ray radiosensitization efficacy was tested in a 3D cell model (spheroids), which represents a step toward real tumor models in vivo. In this experiment, the PC3 cells were cocultured for 24 h with the Au‐Fe‐B NPs before inducing the 3D growth into spheroids, which were then treated with XRT at different doses (0, 5, 10, 15 Gy) and monitored for up to 11 days (Figure S14). The results showed a clear effect of NPs in reducing spheroids growth after XRT, compared to the 3D cell aggregate without NPs. This difference is especially evident at the lowest dose used (5 Gy). Also, the observation of spheroids morphology evidenced, in the case of the cells treated with the NPs, an irregular shape, the presence of debris and dead cells in the surrounding, more necrotic areas, and an overall reduced recovery capacity of cells that, in fact, stopped their expansion. The observation of spheroids also indicated that the Au‐Fe‐B NPs remained in the tumor cells during the observation period of 11 days.

## 3 Discussion

### 3.1 Prospects and Challenges for the Theranostic Multifunctionality of Au‐Fe‐B NPs

The most intriguing aspect of nanomedicine is the opportunity of combining multiple functions in a single nanometric structure aimed at diagnostic and therapeutic applications [34]. Here, the realization of trimetallic NPs made of Au, Fe, and B was undertaken, aiming at their intrinsic set of theranostic functions, which are the multimodal radiosensitization for XRT and BNCT empowered by the ability of multimodal tracking of the NPs with MRI and CT, concluding with other essential attributes as the easy surface coating with biocompatible molecules and the chemodegradation over time. Most importantly, Au, Fe, and B are well‐tolerated by the organism, the most reactive of the three being iron, which, however, is involved in human metabolism [36, 57, 58]. Overall, the Au‐Fe‐B NPs have multiple functions which are hardly found all together even in the most promising sensitizers for BNCT, including carborane derivatives [77, 78, 79]. Carboranes are unsaturated boron‐containing heterocyclic molecules which offer several advantages for BNCT due to their structural stability, high boron content, good tolerability, versatility, and possibility of conjugation with other functional platforms such as dendrimers, magnetic NPs, metal NPs, nanotubes, carbohydrates, Gd chelates, or lipidic particles [77, 78, 79, 80, 81]. High loading in cancer cells and BNCT efficacy in vitro and in vivo has been demonstrated for carborane derivatives, but further progress is undergoing to simplify the synthesis protocols while also improving the selectivity for tumor tissues and the biocompatibility [77, 78, 80]. Also, combined theranostic applications have been considered, especially by conjugation of carboranes with Gd derivatives, which act as MRI contrast agents, neutron capture therapy sensitizers and, potentially, can absorb X‐rays for XRT, although this last condition has been little explored so far [77, 78, 80].

Consolidating multiple functions into a single NP reduces the need for separate injections of MRI, CT, XRT, or BNCT agents, ensuring that all therapeutic and diagnostic nanoagents undergo the same biodistribution, while also minimizing cumulative dose and complex scheduling of each administration [34]. It also enables sequential or simultaneous operation, starting with diagnostic imaging for tumor area delimitation and then moving to radiotherapy treatment [3, 4, 8]. Thus, the integration of theranostic functions in a single nanomedicine avoids the mismatched pharmacokinetics inherent to separate administrations and improves quantitative dose planning (e.g., boron dosimetry) while simplifying patient logistics and reducing the stress due to repeated administrations [8, 17, 18, 19]. However, the novelty of this integrated multimodal approach has several practical constraints before its translational application. The integration of MRI and CT with XRT planning is well established, especially regarding MRI‐linac equipment [3, 82], which represents state‐of‐the‐art for precise radiation dose delivery. Only when hard tissues must be treated, CT imaging is more appropriate for X‐ray dose planning [26, 33], and in this case, the stages of imaging and irradiation must be performed with distinct instruments, transporting the patient in distinct locations, according to current clinical protocols. In fact, while the molar sensitivity of MRI contrast agents is up to 50 times larger than CT, the two techniques are complementary for the imaging of soft (MRI) and dense (CT) tissues, respectively [26, 33].

Neutron irradiation for BNCT is a more recent approach, which is not currently performed simultaneously to imaging equipment, because it requires specialized reactors or accelerator sites. In this case, transport of the patient and tight coordination with imaging and XRT facilities are required, according to current treatment protocols. For instance, the pioneering investigations on the advantages of combined BNCT and XRT were carried out in distinct locations [22, 23, 24, 25]. When imaging and therapy take place with distinct equipment in distinct locations, the use of a multimodal theranostic agent significantly reduces the time windows for imaging, sensitizer accumulation, and therapeutic irradiation, allowing a faster treatment and the possibility of rapid sequential irradiation with X‐rays and neutron beams, minimizing patient discomfort [4, 11, 18, 34, 83]. Streamlining imaging and therapy steps is useful also in the context of more complex cancer treatment protocols, which include chemotherapy or immunotherapy [2, 5, 34, 84]. Overall, the use of multimodal nanoagents can streamline workflows and enhance precision, but their clinical adoption depends on available instrumentation and the feasibility of coordinating multi‐facility procedures, as well as in the future development of platform which integrates bimodal imaging and bimodal radiotherapy in the same location for maximum rapidity and efficacy.

### 3.2 Structural Insights on Au‐Fe‐B NPs

Despite the favorable set of Au‐Fe‐B NPs characteristics for nanomedicine application, little is known about the structural, synthetic, and thermodynamic challenges undermining the way to their realization. Therefore, density functional theory (DFT) modeling was used to understand the energetics and preferred ordering of the Au‐B‐Fe system. We chose DFT because it is the method of choice to model metallic systems and is an *ab‐initio* method, which means that it does not need prior knowledge of the system's properties, which are unavailable in the case of an unknown trimetallic alloy.

**FIGURE 7:**
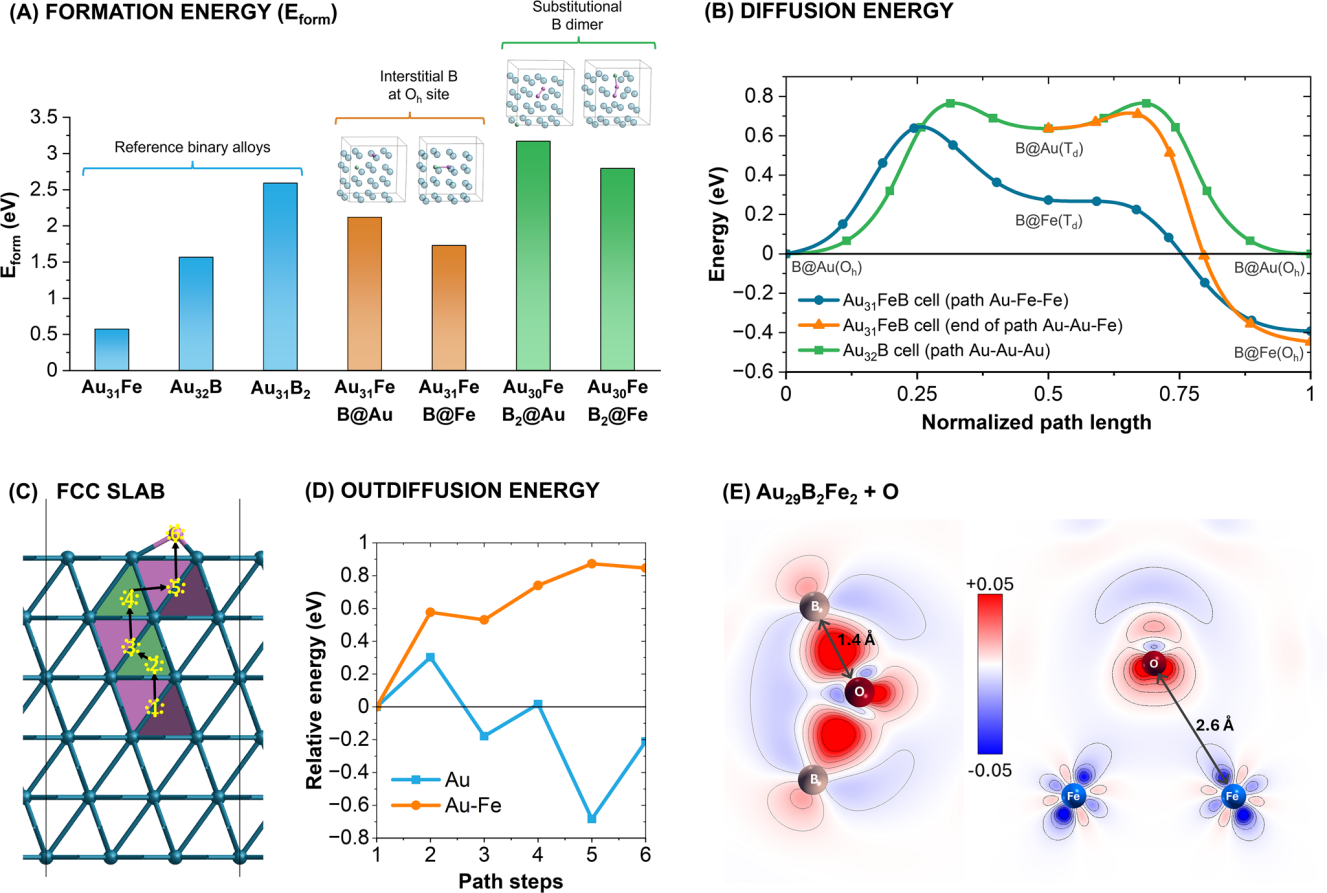
DFT modeling. (A) Formation energies of ternary bulk systems and reference binary systems. The B@Au label indicates models where the boron atom is surrounded by Au only, while the B@Fe label where B is close to Fe. (B) Diffusion of boron: the three curves represent minimum energy paths (symbols indicate the path discretization steps). In all cases, the starting point is an O_h_ site surrounded by Au only, labeled as B@Au(O_h_). Green (Au‐Au‐Au path): diffusion from the starting point to a T_d_ site surrounded by Au (B@Au(T_d_)) and then to another B@Au(O_h_) site. Orange (end of Au‐Au‐Fe path): diffusion from a B@Au(T_d_) site to a B@Fe(O_h_) site. Blue (Au‐Fe‐Fe path): diffusion from the starting point to a T_d_ close to a Fe atom (B@Fe(T_d_)) and then to an O_h_ site close to the same Fe (B@Fe(O_h_)). (C‐D) Outdiffusion of interstitial B. In the slab (C), the purple polyhedra represent O_h_ voids, green polyhedra T_d_ voids. Yellow numbers indicate the intermediates. Relative energies of intermediates (D) for a pure Au slab (blue line) and an Au slab with a Fe channel (orange line) are plotted. (E) Map of the charge density difference projected on a plane passing through B─O (left) and Fe─O (right) bonds in a Au_29_B_2_Fe_2_O model, where O bonds both B and Fe. Fe atoms are blue, B atoms are gray, O atoms are dark red. Red and blue areas indicate regions of electron density buildup and depletion, respectively.

In the first instance, the formation energy (E_form_) of a series of 2 × 2 × 2 FCC supercells describing the Au‐B, Au‐Fe, and Au‐Fe‐B systems was calculated with various configurations (Figure 7A; Table S2). Previous studies for the binary Au‐B alloy indicated that a boron dimer in a substitutional site required a lower formation energy per boron atom than when boron is in an interstitial site, whereas substitutional boron is not compatible with FCC noble metals [8, 85]. Here, the substitutional B impurity was modeled using a bulk Au_31_BFe model, where Fe occupies a substitutional FCC site, and B was placed in interstitial sites either close to Fe (B@Fe) or surrounded by Au only (B@Au). The calculated formation energies of the Au‐B‐Fe system are always positive and systematically larger than those of the binary alloys Au_32_B (B interstitial) and Au_31_Fe (Fe substitutional), the latter being the less instable. This is a very important indication on the out‐of‐equilibrium synthesis conditions needed to produce Au–Fe–B NPs, which must be more stringent than those required for Au–Fe and Au–B counterparts, underscoring the inherent difficulties in achieving this novel nanomaterial even by a non‐equilibrium process as PLAL.

Regarding the atomic arrangement within the Au–Fe–B alloy, in both the B@Fe and B@Au cases, the octahedral interstice (O_h_) is the most favorable, but the E_form_ is 0.39 eV lower when the B atom interacts preferentially with Fe. The case of the boron dimer in a substitutional site was modeled using an Au_30_B_2_Fe cell (Figure 7A; Table S2). In accordance with the interstitial B case, the E_form_ of the boron dimer surrounded by Au only (B@Au) is 0.38 eV larger than the model where the dimer interacts directly with Fe (B@Fe), and exceeds 0.58 eV the E_form_ of the binary alloy Au_31_B_2_ (substitutional B dimer).

Overall, the presence of Fe does not favor the formation of an alloy with Au and B, when compared to the binary alloy counterpart, and the Au‐Fe system resulted much less instable than any other configuration with B. Therefore, the energy barriers toward diffusion were calculated to test the chance of boron segregation outside the Au‐Fe FCC crystalline domains (Figure 7B). The calculations considered the case of interstitial B in an Au_31_FeB cell (blue and orange curves in Figure 7B) and compared it with the Au_32_B system (green curve in Figure 7B). In all cases, the interstitial boron impurity in the FCC matrix B departs from an O_h_ site surrounded by Au only (B@Au(O_h_)) and diffuses by alternating O_h_ and T_d_ sites. The barrier toward diffusion of B is 0.76 eV when moving to a T_d_ close to an Au atom (B@Au(T_d_)) and is lowered to 0.64 eV when the T_d_ is close to a Fe atom (B@Fe(T_d_)). Reading the curve from right to left, one obtains the backward path that, for the Au_31_FeB system, describes the detachment of B from Fe. The overall backward barrier toward the diffusion of B from an O_h_ site close to a Fe atom (B@Fe(O_h_)) to the initial O_h_ site surrounded by Au (B@Au(O_h_)) is 1.0 eV (blue curve in Figure 7B) or 1.2 eV (orange curve in Figure 7B), much larger than the 0.76 eV for the Au_32_B system. Overall, these calculations suggest that B is effectively trapped by Fe instead of undergoing segregation.

Nonetheless, in the Au‐Fe‐B NPs obtained by PLAL and cleaned with citrate buffer and dialysis, boron was found in the form of oxide from the XPS analysis, whereas iron was found both as a metal and an oxide inside the same NP according to various characterization techniques. Therefore, the chance of boron segregation by outdiffusion was investigated further with DFT calculations on a model mimicking the interface of an Au‐Fe alloy with interstitial B (Figure 7C). The model consisted of an FCC slab with a thickness of six atomic layers and terminated with a 111 surface, and considered the two cases of pure Au (blue line in Figure 7D) and Au with a channel of segregated Fe (orange line in Figure 7D, see also Figure S15 for the Fe atoms location) [31]. In the pure Au slab, the relative energies of the intermediates along the outdiffusion path indicated the subsurface O_h_ void as the most stable state of the interstitial B, hence surface segregation is favored. This agrees with recent experimental observations about the stabilization of B atoms at grain boundaries in FCC metals [85]. Conversely, the most stable state for B in the Au slab with the Fe channel is at the slab center, and the energy of intermediates increases almost monotonically as the B atom is moved toward the surface. In fact, the presence of Fe does not provide a driving force for the outdiffusion of metallic boron. Since the segregation of Fe in the Au‐Fe lattice is energetically favored according to our previous study [31], it also promotes the accumulation of B inside the particle. However, the percolative iron paths preferentially undergo corrosion in the Au‐Fe lattice [31], a process which is initiated by the cleaning step with citrate buffer in the S6 synthesis protocol.

Coming back to the XPS evidence that B was oxidized more easily than iron, this is expected according to the higher reactivity with oxygen. Hence, as the last step for the DFT modeling, the oxidation was considered by evaluating the affinity of an oxygen atom with Fe and B in the FCC lattice. To this end, we employed a bulk model Au_29_B_2_Fe_2_ derived from a Au_30_Fe_2_ special quasi‐random structure (SQS), where an Au vacancy close to the Fe dimer was occupied by a B dimer. This model was chosen based on our previous observations that the Au‐Fe system was successfully modeled using SQS [31], while the Au‐B system was better described by B clusters embedded in Au vacancies [8]. For evaluating the binding affinity, the oxygen atom was placed in interstitial sites T_d_ or O_h_. Three options were investigated, namely the O atom close to the B dimer but apart from Fe, the O atom close to Fe but apart from B, and the O atom between B and Fe. Formation energies (see Table S3) show that the least preferred site for oxygen is close to Fe (E_form_ = 2.15 eV), whereas the most stable site is between Fe and B (E_form_ = 0.94 eV), followed by the case of oxygen close to B (E_form_ = 1.21 eV). This is in accordance with the formation enthalpies of B_2_O_3_ and Fe_3_O_4_ oxides, which are −1273.5 and −1120.89 kJ/mol, respectively [86]. A more in‐depth analysis of the most stable system (Figure 7E) confirms that, though O between Fe and B interacts with both elements, the B─O bond is much shorter (1.4 Å) and covalent in nature, while the O─Fe bond is much longer (2.6 Å) and mostly electrostatic. Indeed, because of its relatively large first ionization potential (8.3 eV), B is known for the formation of covalent bonds instead of ionic interactions even with the most electronegative elements [45].

Overall, DFT calculations supported the experimental evidence about synthesis, structure, and functional properties of the Au‐Fe‐B NPs (chemical degradability, cytocompatibility, colloidal stability, cellular uptake), indicating that PLAL generated the trimetallic alloy despite it is thermodynamically less favored than the Au‐Fe and Au‐B binary alloys, but the high affinity of B with oxygen led to its oxidation during and after the synthesis. In particular, the need for NPs free from the amorphous byproducts obtained during PLAL forced to the incubation with citrate buffer, which initiated the oxidation of Fe and B inside the NPs, leading to the final trimetallic NPs constituted by an oxide shell and a porous metallic core with oxide domains. The HRTEM images evidenced that these NPs contain several defects and grain boundaries (Figure 2K). These defects are the starting sites for NPs corrosion, followed by NPs perforation and formation of inner cavities, typically accelerated along the regions of the nanocrystals with higher strain [31, 87, 88]. Hollow structures are formed through a nanoscale Kirkendall effect (NKE) when the corrosion of alloys is mediated by surface oxidation, because oxygen atoms have a much slower diffusion coefficient than metal atoms [31, 87, 88, 89]. The NKE is a different mechanism from the isotropic shrinkage of the NPs occurring by direct dissolution of the metal atoms [19, 54, 90].

The transformation of the NPs surface implies the replacement of the stealth PEG coating with serum proteins [5, 31], facilitating the cell uptake observed in the experiments. This is crucial for radiosensitization, as shown by the decrease in the clonogenic cell survival in samples treated with X‐rays or neutrons after incubation with the Au‐Fe‐B NPs. An increased toxicity was also observed because of the higher uptake of the Au‐Fe‐B NPs compared to Au NPs. In a slightly acidic environment (pH 4.7) and in the presence of ROS, such as in the tumor microenvironment [5], Au‐Fe‐B NPs are chemically degraded generating boric acid, which has been associated with inhibition of cell proliferation, induced apoptosis, and reduced amounts of anti‐oxidants in tumor cells [91]. In fact, the use of NPs resulted in a more effective treatment both with X‐ray and neutron radiation types, and the result is comparable to the effect reported in recent studies for other boron‐based compounds for radiation therapy [92]. This effect was not reversed by the presence of a ferrostatin or ROS inhibitor, indicating that the mechanisms of radiosensitization are not dependent on these factors. In the medium term, the structural degradation and embedding in biological components favor the permeation and the diffusion of the NPs in the tumor tissue, possibly also in the hypoxic regions where the transport of matter happens by diffusion instead of permeation from the vasculature [5]. In fact, the loss of the polymeric shell corresponds to the increase of the diffusion coefficient. In the longer term, the smaller size is expected to facilitate body clearance, fulfilling the ideal requisites for a multimodal radiosensitizer [10, 34, 35, 36]. While this study focused more on the design and assessment of the novel multifunctional Au‐Fe‐B nanoalloy, future in vivo experiments are required to verify critical features as chemical degradation and bimodal therapeutic efficacy toward clinical application.

## 4 Conclusion

In summary, we further expanded the knowledge about the exploitability of multifunctional nanostructures in nanomedicine by developing trimetallic Au‐Fe‐B NPs and assessing their potential as multimodal theranostic agents. We specifically checked the contrast ability for MRI and CT, the radiosensitizer potential for XRT and BNCT, the evidence of long‐term structural instability toward chemodegradation in acidic physiological environment, and their intracellular uptake behavior. The synthesis of the Au‐Fe‐B system is extremely challenging due to unfavorable thermodynamic and tendency to segregation and oxidation of boron, as demonstrated by DFT calculations, and it was possible only by resorting to an out‐of‐equilibrium method as PLAL, and a tailored post‐PLAL cleaning procedure. The list of functions brought by the ternary Au‐Fe‐B NPs allowed adding a new promising item to the nanotoolbox of next‐generation nanomedicine. Future work about in vivo therapeutic validation will be necessary to bridge the gap between materials development and clinical application, in order to explore the exploitability of the Au‐Fe‐B NPs for tumor treatments with complementary therapeutic strategies under the guidance of imaging techniques, possibly leading to more personalized oncological protocols.

## 5 Experimental Section

### 5.1 Synthesis

Au‐Fe‐B NPs were synthesized starting from the PLAL of trimetallic bulk targets placed at the bottom of a cell closed with a transparent glass lid and containing the liquid solution containing the PEG derivatives [8, 19, 31]. The cell was mounted on a motorized XY scanning stage (Standa) controlled with a 2‐axis stepper and a DC motor controller. Laser pulses of a Q‐switched Nd‐YAG laser (1064 nm, 6 ns, 50 Hz) were focused on the target at a fluence of 15 J/cm^2^ by a lens with focal length f = 100 mm. During the laser synthesis, the liquid was kept under Ar flux and stirred with a Teflon‐coated magnetic bar. In S1 (ethanol) and S2 (acetone), a bulk target with composition Au‐Fe‐B 33.3‐33.3‐33.3 at% (99.9% pure, from Mateck GmbH) was used. In S3 (acetone), a bulk target with composition Au‐Fe‐B 15‐33‐52 at% (99.9% pure, from Innovative Materials s.r.l.) was used. In S4 (ethanol) and S5 (acetone), a bulk target with composition Au‐Fe‐B 25‐60‐15 at% (99.9% pure, from Mateck GmbH) was used. Ethanol (HPLC grade, from Sigma–Aldrich) and acetone (anhydrous, VWR) were used as received without further purification. In S1‐5, PEG‐SH (5000 Da, Laysan Bio) was dissolved in the organic solvents at 0.087 mg/mL. For the PEG‐Au‐Fe‐B NPs S6, the S5 procedure was modified by replacing PEG‐SH with a 1/1 w/w mixture of NH_2_‐PEG‐SH (5000 Da, Laysan Bio) and COOH‐PEG‐SH (3000 Da, Rapp Polymere), dissolved in the organic solvents at a total polymer concentration of 0.087 mg/mL.

After PLAL, the colloid was stored at −20°C overnight, and the NPs were collected by centrifugation for 1 h at 1000 rcf and 5°C. The procedure of storage at −20°C overnight and centrifugation was repeated three other times with a mixture of methanol and ethanol (1/4 v/v, both HPLC grade, from Sigma–Aldrich) to remove unbound PEG‐SH and any other synthesis byproduct. Finally, the NPs were dried with a nitrogen flux at room temperature. At this point, the NPs from S1‐5 were resuspended in milliQ water with a bath sonicator (10 min sonication). Instead, the PEG‐Au‐Fe‐B NPs from S6 were suspended in a water solution of PEG‐SH (5000 Da, 3 mg/mL in milliQ water) with a bath sonicator. After 10 min of sonication, the colloid was mixed with citrate buffer (pH 4.7, final concentration of 0.352 mg/mL of citric acid and 0.442 mg/mL of sodium citrate dihydrate in milliQ water) and aged for 30 min at room temperature. Then, the solution was washed 4 times by dialysis at room temperature with milliQ water using concentration membranes (1 h at 500 rcf in Sartorius tubes, 10 kDa cut off) until obtaining the final colloid S6 in water.

The S6 formulation was stored in the fridge (4°C) prior to other experiments. The colloidal stability with this storage condition was assessed by DLS (initial hydrodynamic size 42 ± 11 nm, PDI 0.069; after 1 month 44 ± 11 nm, PDI 0.062), confirming that no changes took place in water at 4°C for a storage up to 1 month. Similarly, no changes were detected in the UV–vis absorption spectra of the S6 sample stored for 1 month.

Reference PEG‐coated Au NPs were synthesized starting from the PLAL of a bulk Au target (99.99% pure) in distilled water containing NaCl 10^−4^ m, using the same set‐up parameters as the S1‐6. Subsequently, PEG‐SH was added at a final concentration of 0.1 mg/mL, and the mixture was incubated overnight at room temperature. Finally, the PEG‐Au NPs were washed by dialysis with milliQ water until resuspension in pure milliQ water.

### 5.2 Characterization

UV–vis spectra were recorded with a JASCO V770 spectrophotometer using quartz cells with a 2 mm optical path or, for a quick evaluation of PLAL synthesis progress, by an Avantes portable spectrometer (AvaSpec‐ULS2048CL‐EVO) coupled with a deuterium‐halogen lamp (AVA‐Light‐DHc) and a peek‐coated optical fiber probe. DLS was performed with a Malvern Zetasizer Nano ZS in DTS1070 cells. FTIR spectroscopy was performed with a Perkin Elmer 1720X spectrophotometer, depositing the dried samples on a KBr window.

EDX analysis on the PEG‐Au‐Fe‐B NPs drop cast on a crystalline Si substrate was performed with an ESEM model FEI Quanta 200, without further metallization.

Sample elemental composition and concentration were assessed with ICP‐MS using an Agilent Technologies 7700x ICP‐MS (Agilent Technologies International Japan, Ltd., Tokyo, Japan). The instrument was equipped with an octupole collision cell operating in kinetic energy discrimination mode for the removal of polyatomic interferences and argon‐based interferences. Samples digestion was performed with aqua regia at 100°C for 1 h. For instrument calibration, standard solutions were IV‐ICPMS‐71A Multielement calibration standard (Inorganic Ventures, 100 mL solution containing 10 mg/L each of Ag, Al, As, B, Ba, Be, Ca, Cd, Ce, Co, Cr, Cs, Cu, Dy, Er, Eu, Fe, Ga, Gd, Ho, K, La, Lu, Mg, Mn, Na, Nd, Ni, P, Pb, Pr, Rb, S, Se, Sm, Sr, Th, Tl, Tm, U, V, Yb, Zn; a matrix 3% HNO_3_) and the IMS‐103 Multielement calibration standard (Ultra‐scientific multi‐standards, 100 mL solution containing 10 mg/L of Sb, Au, Pt, Rh, Hf, Ru, Ir, Te, Pd, and Sn; matrix 10% HCl/1% HNO_3_).

XRD patterns were collected from powder samples deposited at room temperature on Si zero‐background substrates with a Panalytical XPert 3 powder diffractometer equipped with a Cu tube (40 kV, 40 mA), a BBHD mirror, a spinner, and a PlXcel detector. Crystalline phase identification and Rietveld analysis were executed with TOPAS Academic V6 (Bruker AXS) and ISCD (005‐3764 for Au, 040–1295 and 005–6758 for Au‐Fe) or COD (4003012 for Fe‐B, 9007644 for Fe_3_O_4_) databases. Rietveld refinements were carried out by fitting the background with a Chebyshev function and the required phases. The shape of the reflections was modeled through the fundamental parameter approach incorporated in the program, separating the instrumental and the sample contributions. Fit indicators R_wp_, R_exp_ and GoF were used to assess the quality of the refined structural models.

XPS spectra were acquired in an ultrahigh vacuum (UHV) chamber operating at a working pressure below 5 × 10^−8^ mbar. Samples were drop‐cast at room temperature on glassy carbon substrates. The measurements were performed with Al Kα radiation (hν = 1486.7eV) and Mg Kα radiation (hν = 1253.6 eV), each time on a freshly deposited sample, with pass energies of 50 eV for survey and B 1s spectra and 20 eV for high‐resolution spectra. Sputtering was performed at 1.5 kV for 5 min with Ar^+^ ions. The relative composition of B and Au was determined by normalizing the intensity of their respective photoemission lines, acquired under identical conditions (pass energy 50 eV), using the respective sensitivity factors. To calculate the ratio of metallic and oxide components, the Fe 2p_3/2_ peak was deconvoluted using XPS Peak 4.1 software, as described in Ref. [93].

Bright field TEM analysis was performed with a FEI Tecnai G2 12 operating at 100 kV and equipped with a TVIPS CCD camera. HRTEM, SAED, STEM‐EDX, and EELS analyses were performed with a TEM Talos F200S G2 operating at 200 kV and equipped with two windowless silicon drift X‐ray detectors and a Gatan Infinium SE/976 spectrometer. EELS spectra were corrected for instrumental background, and a single‐exponential fit was applied to subtract the contribution due to the tail of the zero‐loss peak. For each sample, a drop of the solution was deposited on a copper grid coated with an amorphous carbon film. ImageJ software was used to measure the geometrical (Feret) size distributions. The average Feret size and standard deviation were obtained from statistics considering more than N = 500 NPs for each sample.

### 5.3 Chemical Degradation of NPs

For PEG‐Au‐Fe‐B NPs transformation over time in biological fluids, the NPs were dispersed at 0.05 mg/mL in 20% v/v FCS at pH 7.4, 6.5 (by adding citrate‐phosphate buffer) or 4.7 (by adding citrate buffer), incubated at 37°C, and analyzed at different time points from 0 to up to 2 months. At each time point, UV–vis analysis was performed, and a drop of the solution (diluted 1:9 v/v with milliQ water) was deposited on a copper grid coated with an amorphous carbon film. Statistics considered more than 500 NPs for each sample, using the ImageJ software. At the end of the 2‐month incubation period, the NPs were collected by centrifugation at 10 000 rcf for 30 min, deposited on Si zero‐background substrates, and analyzed by XRD.

### 5.4 MRI

Magnetic resonance images of NPs phantoms were acquired using a Bruker system operating at 7 T (Bruker Biospin, Ettlingen, Germany). In phantom measurements, the samples were dispersed in milliQ aqueous solution by serial dilution starting from a solution with an Fe concentration of 1.30 mm. The transversal relaxivities (r_2_ values) were calculated from the slopes of the best‐fit lines of relaxation rates (1/T_2_) vs. iron concentrations. The T_2_ map phantom images were acquired using a multislice multiecho sequence with the following parameters: TR = 2000 ms, TE = from 6.5 to 170.43 ms, FOV = 55 × 55 mm, matrix size = 128 × 128, slice thickness = 1 mm, number of echoes = 25.

To evaluate in vivo the biodistribution of Au‐Fe‐B NPs, C57/BL6 male mice (6 weeks old, N = 3, Envigo) were intravenously injected with NPs at a dosage of 150 mg/kg (200 µL, 44 mg Fe/kg, 104 mg Au/kg) in the mouse tail vein. MRI studies were performed on a 7‐T Preclinical Scanner (BioSpec 70/30 USR, Paravision 6.0.1; Bruker) equipped with 450/675 mT/m gradients (slew rate: 3400–4500 T/m per second; rise time: 140 µs) and a circular polarized mouse body volume coil with an inner diameter of 40 mm.

For each MRI exam, a mixture of IsoVet (isoflurane 1%–2%) with oxygen was used to anaesthetize animals, and the breath rate was constantly monitored to regulate the level of anaesthesia. Morphological images of the liver, kidneys, and spleen were acquired using Multi Slice Multi Echo (MSME), Rapid Acquisition with Relaxation Enhancement (RARE) T_2_‐weighted sequences with the following relative parameters: for MSME, (TR) = 2600 ms, (TE1) = 7.02 ms and echo spacing = 7.02 ms, spatial resolution = 0.175 × 0.138 mm/pixel, scan time = 7 min; for RARE T_2_, TR = 3500 ms, TE = 30 ms, spatial resolution = 0.175 × 0.122 mm pixel. All the sequences were acquired with the same field of view (FOV = 35 × 22 mm) and thickness (1 mm). The images were acquired before, 1 h and 24 h after NP injection. In vivo experiments were carried out with the authorization of the Italian Ministry of Health and the Committee for Research on Laboratory Animals of the San Raffaele Hospital (847/2023‐PR).

### 5.5 CT

CT images of NPs phantoms were acquired using a small‐animal CT scanner (x‐rad, SmART, Precision X‐ray) using the following acquisition parameters: tube tension 80 kVp, current 3 mA, 300 views, 0.1 mm voxel size. Images were reconstructed using the Feldkamp algorithm for cone beam CT. In phantoms, the samples were dispersed in 1 w% agarose solution by serial dilutions, starting from a concentration of 4.97 mg/mL in Au. X‐ray attenuation ability in HU mL/mg_Au was calculated from the slopes of the best‐fit lines of HU vs. gold concentration.

In vivo CT images were acquired using a dedicated small‐animal CT scanner (X‐rad, SmART, Precision X‐ray) using the following acquisition parameters: tube tension 80 kVp, current 3 mA, 300 views, 0.1 mm voxel size. CT image acquisitions in vivo were performed before and at 0 h, 24 h, 4 days, 8 days, and 14 days after injection. During image acquisition, the animals were kept at 37°C and under gaseous anaesthesia (2%−3% isoflurane and 1 l/min oxygen). No weight loss or signs of suffering were detected in mice over the experiment. Image analysis (using imageJ) was performed by placing five different regions of interest (ROI) on the corresponding organs (liver, spleen, and kidneys), and the mean HU value of each ROI was calculated. In vivo experiments were carried out with the authorization of the Italian Ministry of Health and the Committee for Research on Laboratory Animals of the San Raffaele Hospital (847/2023‐PR).

### 5.6 Neutron Autoradiography

The neutron capture by ^10^B isotopes in the Au‐Fe‐B NPs was evaluated by neutron autoradiography [8, 19, 94]. The Au‐Fe‐B NPs were deposited by serial drop casting on CR39 solid‐state nuclear track detectors and dried at room temperature. The CR39 films were irradiated in the thermal column of the TRIGA Mark II reactor. For quantitative track counting analysis, the reactor operated at 2 kW for 30 min, with a neutron fluence of (2 ± 0.1) × 10^10^ cm^−2^. Then, the CR39 film was etched with PEW40 solution, imaging the tracks generated by the charged particles emitted from the ^10^B atoms with a LEICA M205 FA stereo microscope, and counting the number of tracks per drop using the Scikit‐Image blob detection algorithm [8, 19, 94]. For qualitative imaging of charged particle tracks, the reactor operated at 250 kW for 2 h, obtaining a neutron fluence of 9.8 ± 0.5 × 10^12^ cm^−2^. Then, the images of the dried drops were obtained by etching with sodium hydroxide the CR39 slides and collecting the images with the stereo microscope.

### 5.7 Cytocompatibility

Cellular viability was assessed with the 3‐(4, 5‐dimethyl‐thiazol‐2‐yl)‐2, 5‐diphenyl‐tetrazolium bromide (MTT) assay (Sigma–Aldrich). BJ fibroblasts (ATCC cat number CRL‐2522) were seeded in a 96‐well plate at a density of 5 × 10^3^ cells/well, PC3 (ATCC, CRL‐1435) and HEK (ATCC, CRL‐1435) cells at 3 × 10^3^ cells/well, then left in the incubator at 37°C and a 5% CO_2_ atmosphere overnight. After this period, the medium was removed, and 100 µL of fresh medium with NPs was added to each well and incubated for 24h or 48h. PEG‐Au‐Fe‐B NPs or PEG‐Au NPs were added to each well from a stock solution, with a maximum dilution of the culture medium of 10% in volume at the highest NPs concentration of 300 µg/mL. For the ferroptosis experiments, mirrored wells w/o and with NPs at 50 µg/mL were co‐incubated with ferrostatin‐1 (Fer‐1, Sigma–Aldrich) at 2 µm for 24 h and compared with the groups without Fer‐1. After the incubation period, wells were washed with PBS, and MTT was added at a final concentration of 0.5 mg/mL. After 2 h of incubation, the MTT solution was removed, and the formazan crystals were dissolved in 100 µL of dimethyl sulfoxide. Absorbance was read at 590 nm in a multiplate reader. Viability was calculated as a ratio of the absorbance of the well divided by the mean absorbance of the control group. Each condition was prepared in triplicate, and at least three independent experiments were performed.

For NPs uptake quantification by ICP‐MS, PC3 cells were plated at a concentration of 3 × 10^5^ cells/well and in 2 mL of medium, one well per group, in a 6‐well plate and left in the incubator overnight. Complete medium with NPs was prepared at concentrations of 50 and 100 µg/mL, depending on the groups, at a final volume of 1 mL in each well. After 24 h of incubation, the medium was removed, wells were washed with PBS, and 0.2 mL of trypsin/EDTA was added until cells were detached. After the addition of 1 mL of medium to the wells, cells from each group were counted manually with a hemocytometer, then centrifuged for 5 min in 1.5 mL conical tubes. The supernatant was then removed, and the cells were resuspended in 1 mL of PBS. Three independent experiments were performed. ICP‐MS analysis was carried out after digestion of cell samples with di HNO_3_ (69 wt.%, analytical grade) and HCl (37 wt.%, analytical grade) at a 0.25/0.50 w/w ratio at 100°C for 2 h.

For SEM analysis, PC3 cells were plated at a concentration of 0.5 × 10^5^ cells/well and 2 mL of medium, one well per group, in a 24‐well plate containing 18 mm borosilicate glass slides (thickness 0.16–0.19 mm, sterilized), and left in the incubator for 2–3 days until fully attached. NPs were incubated for 24 h in complete medium at a volume of 0.4 mL. Then, the NPs medium was removed, and slides were washed 2× with PBS before the addition of 200 µL of PFA 4% and incubation at room temperature for 40 min to fix the cells. Then, slides were washed with PBS and mounted on a microscope glass slide using a drop of glue (Dako), with cells facing up. SEM images and EDX maps of cells were collected with the ESEM without further metallization. Images were collected both in Z‐contrast (Z‐BSE) and Topography (TOPO) mode using the backscattered electrons detector (dualBSD). Image segmentation was performed with an ImageJ plug‐in (“Trainable Weka Segmentation”) [95], using at least four representative images for each sample and under human supervision for each image.

For the detection of intracellular hydrogen peroxide, PC3 human prostate cancer cells were cultured in RPMI‐1640 medium supplemented with 10% (v/v) fetal bovine serum (FBS) and 1% (v/v) Penicillin‐Streptomycin (Pen‐Strep), under standard culture conditions (37°C, 5% CO_2_, humidified atmosphere). Then, 6 × 10^3^ cells/well were seeded in white opaque 96‐well plates and allowed to adhere for 24 h. Cells were then treated with Au‐Fe‐B NPs at a final concentration of 50 µg/mL for 24 h. Following nanoparticle treatment, cells were washed with PBS and treated with X‐ray at doses of 2 Gy and 4 Gy. Two experimental groups were analyzed: cells treated with XRT alone and cells treated with NPs + XRT, at both radiation doses. The ROS‐Glo H_2_O_2_ Assay (Promega, TM391) was adopted: briefly, 20 µL of ROS‐Glo Detection Solution was added directly to the cell media and incubated for 6 h at 37°C. After incubation, ROS levels were quantified by adding the ROS‐Glo Luciferin Detection Reagent and measuring luminescence using a microplate reader (e.g., VICTOR Nivo, PerkinElmer). All conditions were tested in independent triplicates. Luminescence values were expressed in relative light units (RLU) and used to compare ROS production between experimental groups.

For the GSH/GSSG assay using the GSH/GSSG Ratio Detection Assay Kit II – Fluorometric (Green) (ab205811, Abcam, UK), 6 × 10^3^ cells per well were seeded into black‐wall 96‐well plates with clear bottoms and allowed to adhere for 24 h. Cells were then treated with Au‐Fe‐B NPs at a final concentration of 50 µg/mL for 24 h. Following nanoparticle treatment, cells were washed with PBS. Cells were lysed in the provided lysis buffer, deproteinized using the supplied Deproteinizing Sample Preparation Kit (TCA‐based), and fluorescence intensity was measured at Ex/Em = 490/520 nm using a microplate reader (VICTOR Nivo, PerkinElmer). All measurements were performed in independent triplicates, and results were expressed as GSH/GSSG ratios calculated from fluorescence values based on a standard curve prepared using the kit's glutathione standards.

### 5.8 XRT

For in vitro X‐ray irradiation studies, the clonogenic assay was used to evaluate cell survival. PC3 cells were seeded at 4 × 10^3^ cells/well in quadruplicate in 96‐well plates and left overnight in the incubator to grow, then the NPs were added at 50 or 100 µg/mL in 100 µL of medium/well. After a 24 h incubation period, wells were washed with PBS, and fresh media were added. For evaluation of the ROS scavenger effect of catalase (CAT, Sigma–Aldrich) and ferroptosis inhibition effect of Fer‐1, freshly prepared solutions in medium were added to wells after media removal in a volume of 50 µL, then, after 1 h, 50 µL of medium with NPs (50 µg/mL) was added to each well resulting in a final concentration of 500 µg/mL for CAT and 2 µm for Fer‐1. Then, plates were irradiated at doses from 0 to 8 Gy, with each dose prepared in a separate plate, in a small animal irradiator (SmART system, Precision Xray, USA) at 225 kV, 13 mA, using a 2 mm copper filter and 4 Gy/min dose rate. After irradiation, cells were detached from the plates using a standard protocol with trypsin, and each condition was counted separately using a hemocytometer. Then, each group was plated in triplicate with 400 cells/well in a 6‐well plate for the 0 and 2 Gy, and at 2×, 4×, and 10× cell concentration for 4, 6, and 8 Gy, respectively, using 2 mL of medium per well. After 8 days in the incubator, the plates were coded for a blinded count, then colonies were stained after medium removal using 0.5 mL/well of a crystal violet solution (0.1% crystal violet, 11% ethanol). After 5 min, the staining solution was removed, plates were rinsed by immersion in water, and left to dry at room temperature. Colonies were then counted, and the relative survival fraction was calculated using the standard formula for survival fraction (SF) = (number of colonies)/(number of cells seeds × plating efficiency (PE)), in which PE = (number of colonies counted / number of cells plated).

For XRT on 3D cell cultures, spheroids (with or without nanoparticle treatment) were generated from PC3 cells seeded in quadruplicate at a density of 6 × 10^3^ cells per well in flat‐bottom 96‐well plates and allowed to adhere for 24 h. The following day, Au‐Fe‐B NPs were added at a final concentration of 50 µg/mL in 100 µL of culture medium per well. After 24 h of incubation, cells were detached, centrifuged, and counted in order to seed 2.5 × 10^3^ cells/well for spheroid formation. Geltrex basement membrane matrix was added to the cell suspension at a final concentration of 1% (v/v), and 200 µL of this mixture was dispensed into each well of ultralow attachment (ULA), round‐bottom 96‐well plates (Corning). The plates were centrifuged at 1, 500 rpm for 10 min at 4°C to promote cell aggregation and subsequently incubated under standard conditions. By day 3, compact and uniform spheroids had formed and were subjected to X‐ray radiation treatment. Irradiation was performed using a small animal image‐guided irradiator (SmART system, Precision X‐Ray, USA) at a voltage of 225 kV and a current of 13 mA, with a 2 mm copper filter and a dose rate of 4 Gy/min. Spheroids were irradiated at doses of 5, 10, or 15 Gy. Following irradiation, all spheroids were maintained in culture under standard conditions. To monitor spheroid growth over time, bright‐field images were acquired at 5× magnification using an inverted microscope (Axio Observer.Z1, Carl Zeiss Microscopy GmbH, Germany) from day 0 to day 11. For each spheroid and time point, the average diameter was measured using ImageJ (NIH, USA) to assess growth dynamics over time across treatment conditions.

### 5.9 Neutron Irradiation of Cell Cultures

For the in vitro BNCT experiments, PC3 cells were grown in 96‐well plates at 37°C humidified air (5% CO_2_), in RPMI 1640 (EuroClone S.p.A.), supplemented with 10% v/v FBS (EuroClone S.p.A.) and 1% Penicillin‐Streptomycin (EuroClone S.p.A.). The irradiation was performed on continuously growing non‐confluent cell populations by seeding cells 48 h before exposure to the NPs at a density of 6 × 10^3^ in ten wells of 96‐well plates. Cells were incubated for 24 h with 100 µg/mL (final concentration) of PEG‐Au‐Fe‐B NPs. After 24 h, the medium with NPs was removed, cells were washed three times with PBS (Euroclone S.p.A.), fresh medium was added, and the plates were subjected to neutron irradiation at 50, 100, 200, or 250 kW of irradiation power in the TRIGA Mark II reactor. Cell samples were prepared in a separate plate for each irradiation power. The reactor power and the irradiation times were set to deliver absorbed doses of 1, 2, 4, and 6 Gy for the non‐treated control.

For survival rate evaluation, performed by the plating assay, after serial dilutions, cells were plated at three different concentrations (range 50–5000 cells per Petri dish) in five replicate Petri dishes and allowed to grow at 37°C for 8 days. Subsequently, colonies were fixed in 70% ethanol, stained with Toluidine blue, and counted. The ratio between the number of colonies with more than fifty cells and the number of seeded cells gave the value of the plating efficiency (PE). The cell survival fraction (SF) was obtained by dividing the PE of the treated samples by the PE of the control sample.

To assess the presence of boron inside cells, in vitro neutron autoradiography was employed by adapting the method described in ref. [96]. In vitro autoradiography was performed by culturing PC3 cells on a solid‐state nuclear track detector (polycarbonate Lexan). First, the detector was incubated overnight with culture medium, then PC3 cells were seeded at 5 × 10^4^ cells for 3 mL of medium (value optimized on the basis of cell adhesion tests at different cell densities). Cells were grown for 24 h, then the medium was replaced with a fresh one containing the Au‐Fe‐B NPs at 50 µg/mL and incubated for an additional 24 h. Then, cells have been washed three times with PBS and fixed for 20 min at room temperature with glutaraldehyde 2.5% in NaCacodylate:H_2_O 1:1. After fixation, cells were washed three times with water and once with ethanol and left to dry at room temperature. The fixed cell samples were irradiated with neutrons for 2 h at 250 kW (10^13^ neutrons/cm^2^) in the TRIGA Mark II reactor. For tracks identification on Lexan, cells were stained for 15 min with hematoxylin, washed thoroughly with water, and dried for 24 h at room temperature. Finally, the samples were exposed to UV‐C for 5 min for development and etched in PEW40 at 70°C for 4 min. The ROIs were analyzed with optical microscopy in direct and reflected light at various magnifications (5, 10, 20, 50 ×).

### 5.10 DFT Calculations

Periodic DFT calculations were performed within the plane wave‐pseudopotential approach as implemented in Quantum‐Espresso [97]. The PBE approximation to the exchange‐correlation functional was employed, and ultrasoft pseudopotentials from the GBRV library [98] were used. Wavefunctions were expanded over a plane wave basis set with a cutoff of 40 Ry, while the cutoff on electron density was 400 Ry. Minimum energy paths for the diffusion of boron were obtained using the Climbing Image Nudged Elastic Band (CI‐NEB) approach [99], where paths were converged within 0.05 eV/Å.

Bulk models were 2 × 2 × 2 supercells of face‐centred cubic structures. Variable‐cell relaxation was performed on these models until pressure was lower than 0.5 kbar and forces on ions were lower than 10^−3^ Ry/Bohr. A Monkhorst‐Pack 6 × 6 × 6 k‐points set centered at the Γ‐point was used. Slab models were cut parallel to the (111) plane and were composed of 6 atomic layers, where repeated images were separated by ∼17 Å of vacuum. A 3 × 3 × 1 k‐points integration scheme was used.

### 5.11 Statistical Analysis

In the assessment of average NPs size from TEM measurement, all values were given as mean ± standard deviation (SD) obtained from the size of a minimum of 500 distinct particles, independently measured with ImageJ without any filtering or alteration of the original image. Preprocessing of MRI and CT data was performed by searching for outliers (none found). Linear fitting was performed with no fixed parameters. The slope and the standard error of the linear fit of CT and MRI contrast efficiency were calculated using the least‐squares method of linear regression. In the in vitro biocompatibility assays, all values were given as mean ± SD obtained from three independent experiments (biological replicates), where each experiment was performed with three technical replicates per condition. The interquartile range (IQR) method and a rule of 1.5xIQR were used to identify outliers, which were removed from the dataset if found. Data from in vitro clonogenic survival analysis of each independent irradiation experiment were analyzed in terms of the ratio between treated/control, in which the control was sham‐irradiated and not incubated with NPs. The means ± SD of four independent experiments are shown as the results. In all cases, the normality tests were performed prior to the one‐way ANOVA analysis (Tukey's comparisons test), where the dataset did not reject normality, and to the Kruskal–Wallis ANOVA test, where data rejected normality to identify statistical significance among data with *p* = 0.05. All processing was performed using Origin software (OriginLab, USA).

## Funding

The authors acknowledge the support from AIRC under MFAG 2021 – ID. 25681 project – P.I. Amendola Vincenzo and MSCA Seal of Excellence @ UNIPD 2023 grant NANOXRT.

## Conflicts of Interest

The authors declare no conflicts of interest.

## Supporting information




**Supporting File**: adhm70921‐sup‐0001‐SuppMat.pdf.

## Data Availability

The data that support the findings of this study are available from the corresponding author upon reasonable request.
