# Supplementary material for: Taming the Immiscibility of Gold, Iron, and Boron to Craft Chemodegradable Nanoparticles for Multimodal Imaging and Radiotherapy
Source: Adv Healthc Mater. 2026 Feb 15;15(16):e05031. doi: 10.1002/adhm.202505031 (PMC13107933; doi:10.1002/adhm.202505031)
Supplement: Supplementary file 1 — Supporting File: adhm70921‐sup‐0001‐SuppMat.pdf. [file ADHM-15-0-s001.pdf]

Supporting Information

Taming the Immiscibility of Gold, Iron, and Boron to Craft Chemodegradable Nanoparticles for Multimodal Imaging and Radiotherapy

*Michael Bissoli, Clara M.G. de Faria, Veronica Torresan, Maria Assunta Lacavalla, Mattia Cattelan, Denis Badocco, Paolo Pastore, Pasquina Marzola, Laura Cansolino, Cinzia Ferrari, Ian Postuma, Riccardo Vago, Silva Bortolussi, Antonello E. Spinelli, Daniel Forrer, Vincenzo Amendola***

Mr. Michael Bissoli, Dr. Clara M.G. de Faria, Dr. Veronica Torresan, Dr. Maria Assunta Lacavalla, Prof. Mattia Cattelan, Prof. Denis Badocco, Prof. Paolo Pastore, Dr. Daniel Forrer, Prof. Vincenzo Amendola
Department of Chemical Sciences, University of Padova, Padova, Italy
E-mail: vincenzo.amendola@unipd.it

Dr. Veronica Torresan
Department of Industrial Engineering, University of Padova, Padova, Italy

Prof. Pasquina Marzola
Department of Engineering for Innovation Medicine, University of Verona, Verona, Italy

Dr. Laura Cansolino, Dr. Cinzia Ferrari,
Department of Clinical Surgical Sciences, Integrated Unit of Experimental Surgery, Advanced Microsurgery and Regenerative Medicine, University of Pavia, Pavia, Italy

Dr. Laura Cansolino, Dr. Cinzia Ferrari, Dr. Ian Postuma, Prof. Silva Bortolussi
INFN (National Institute of Nuclear Physics), Unit of Pavia, Pavia, , Italy

Dr. Riccardo Vago
Urological Research Institute, Division of Experimental Oncology, IRCCS San Raffaele Scientific Institute, Milan, Italy

Prof. Silva Bortolussi

Department of Physics, University of Pavia, Pavia, Italy

Dr. Antonello E. Spinelli
Experimental Imaging Center, IRCCS San Raffaele Scientific Institute, Milan, Italy

Dr. Daniel Forrer
CNR – ICMATE, Padova, Italy

Contents

**Figure S1**	***Additional TEM images of S5 and S6 samples***	pg. S3
**Figure S2**	***Additional XPS results with Mg Kα source***	S4
**Figure S3**	***Structural evolution of Au-Fe-B NPs in FCS at pH 6.5 and 4.7***	S5
**Table S1**	***Percentage of ultrasmall (< 5 nm) NPs at various timepoints***	S5
**Figure S4**	***EDX of ultrasmall NPs***	S6
**Figure S5**	***CT data vs time***	S7
**Figure S6**	***Cytotoxicity boxplots and SD-SE plots***	S8
**Figure S7**	***Cytotoxicity vs Au, Fe and B content in Au-Fe-B NPs***	S8
**Figure S8**	***XRT clonogenic assay sketch and images***	S10
**Figure S9**	***BNCT clonogenic assay sketch and images***	S11
**Figure S10**	***XRT clonogenic assay images with Fer-1 and Cat***	S12
**Figure S11**	***Intracellular hydrogen peroxide assay***	S13
**Figure S12**	***GSH/GSSG assay***	S13
**Figure S13**	***In vitro neutron autoradiography***	S14
**Figure S14**	***XRT in 3D cell model***	S15
**Table S2**	***Formation energies and structure of Au-B-Fe models***	S16
**Table S3**	***Formation energies and structure of the $Au_{29}B_2Fe_2$ model with O***	S17
**Figure S15**	***Side view of the 111 slab with Fe channel.***	S18
	Bibliography	S19

Figure S1. ***Additional TEM images of S5 and S6 samples.*** TEM images of S5 (A) and S6 (B) samples, showing the removal of the amorphous matrix surrounding the S5 NPs after the purification procedure reported in Figure S2A. In particular, the incubation with citrate buffer at mildly acidic pH (4.7) for 30 minutes has been previously described as a viable approach to remove amorphous compounds while preserving biodegradable NPs made of iron and boron (Ref.19 of the main article). The chemical degradation of the NPs does not happen on this short incubation time but, as shown in Figure 3, it is observed after 2 months (2880 times longer incubation time).

(A) Au-Fe-B NPs S5 **(B) Au-Fe-B NPs S6**

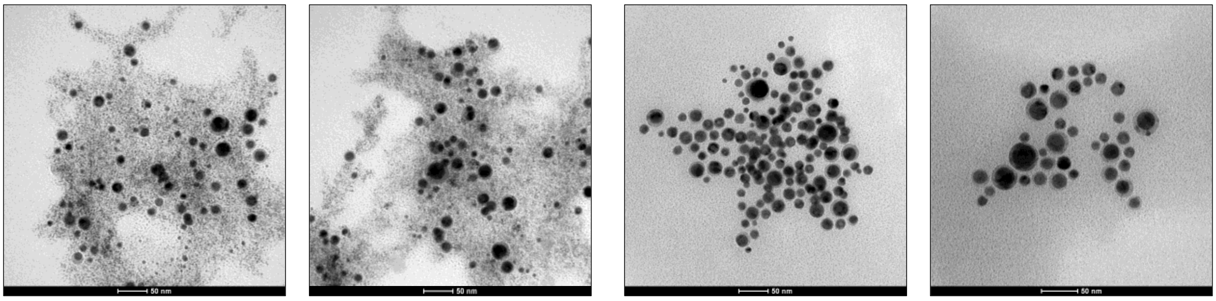

Figure S2. ***Additional XPS results with Mg Kα source.*** XPS analysis of Au 4f (A), B 1s (B) and Fe 2p (C) peaks before (black lines) and after Ar^+ sputtering (red lines) collected with Mg Kα source.

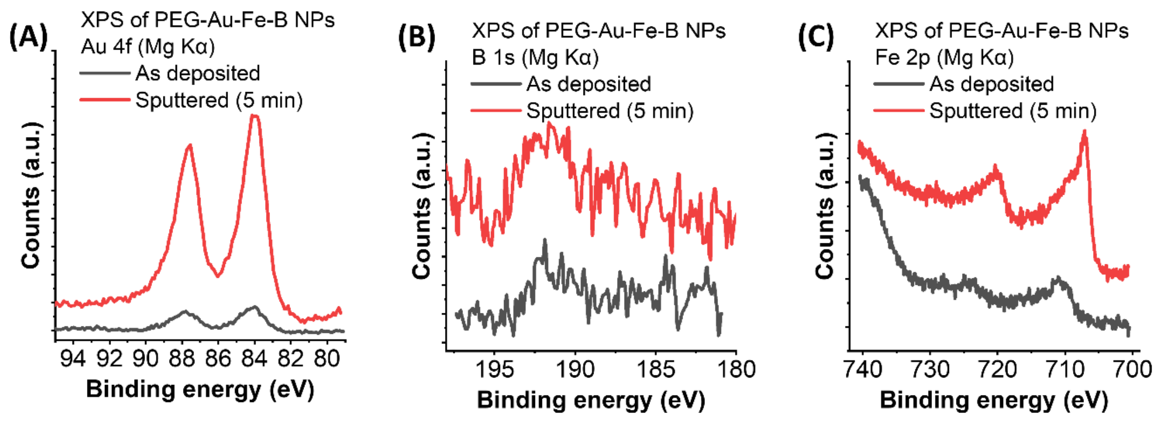

Figure S3. ***Structural evolution of Au-Fe-B NPs in FCS at pH 6.5 and 4.7.*** (A-C) Representative TEM image of the PEG-Au-Fe-B NPs sample before (A) and after incubation in FCS at pH 6.5 (B) and 4.7 (C) for 1-2 months at 37 °C. (D-G) Size distribution and average size with relative standard deviation of the NPs at 0, 1 and 2 months of incubation at pH 6.5 (D-E) and 4.7 (F-G). (H-I) UV-vis at the different incubation timepoints at the two pH. The spectra were normalized at 450 nm for ease of comparison. (J-L) XRD with the corresponding Rietveld fit (grey lines) of the Au-Fe-B NPs after 2 months of incubation at pH 6.5 (J) and 4.7 (K), and at time 0 (L).

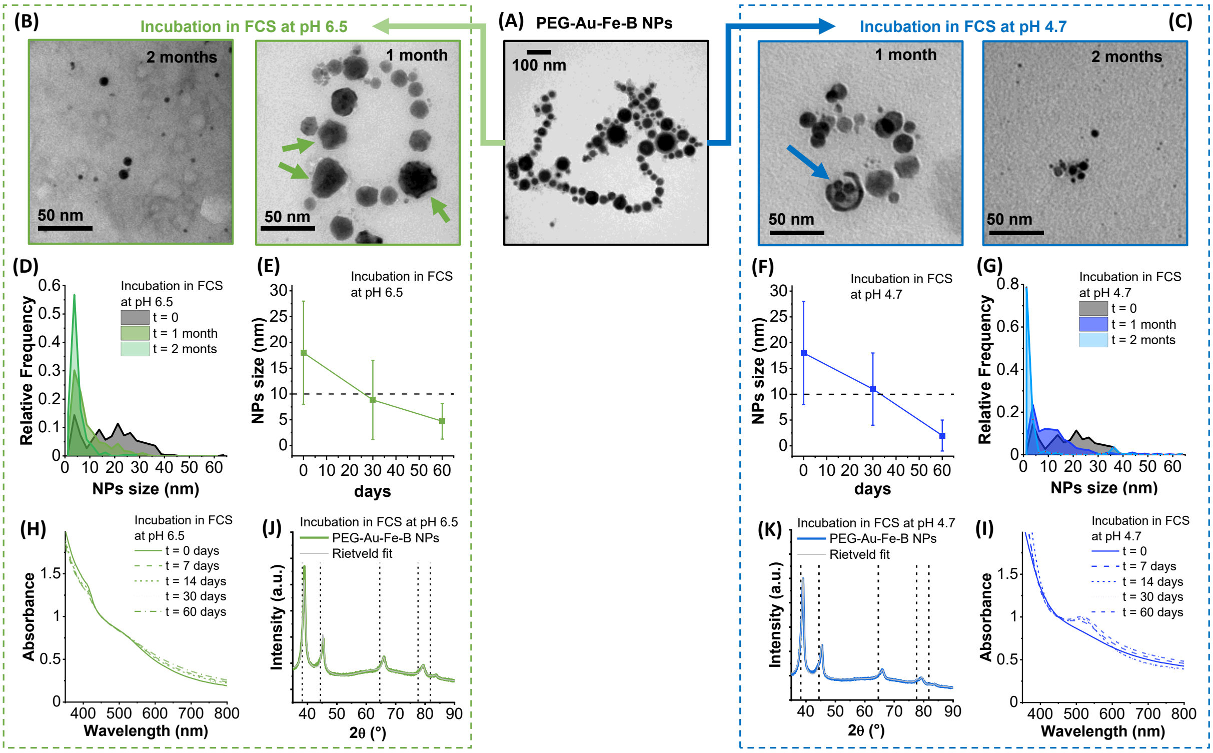

Table S1. ***Percentage of ultrasmall (< 5 nm) NPs at various timepoints.*** The % is reported for the size histograms of the experiments at the three pH levels tested (7.4, 6.5 and 4.7), as obtained from TEM images taken on the PEG-Au-Fe-B NPs samples at 0, 1 and 2 months incubation at 37 °C.

% of NPs < 5 nm	*FCS at pH 7.4*	*FCS at pH 6.5*	*FCS at pH 4.7*
0	15.9 %		
1 month	7.3 %	36.8 %	30 %
2 months	41.9 %	71.6 %	95.4 %

Figure S4. ***EDX of ultrasmall NPs.*** EDX analysis of primary and ultrasmall NPs in the S5 sample. Top: HAADF and net EDX maps of Au, Fe and O. Bottom: EDX quantitative analysis for NPs of size ranging from 30.5 to 4.9 nm, showing similar Au/Fe atomic ratio (at. r.) independent of the size.

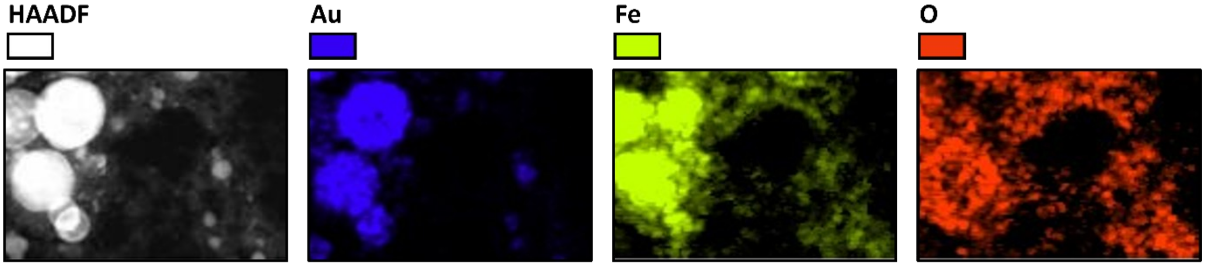

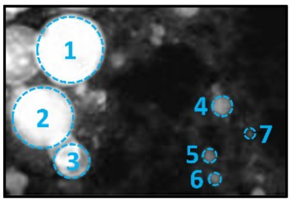

NP #	1	2	3	4	5	6	7
**Size**	30.5 nm	28.0 nm	15.6 nm	9.3 nm	6.3 nm	5.6 nm	4.9 nm
**Au (at. r.)**	0.42	0.31	0.40	0.45	0.64	0.30	0.42
**Fe (at. r.)**	0.58	0.69	0.60	0.55	0.36	0.70	0.58

Figure S5. ***CT data vs time.*** The *in vivo* NPs localization was monitored by CT until 14 days, indicating that the maximum accumulation in the liver and spleen occurred 8 days after administration.

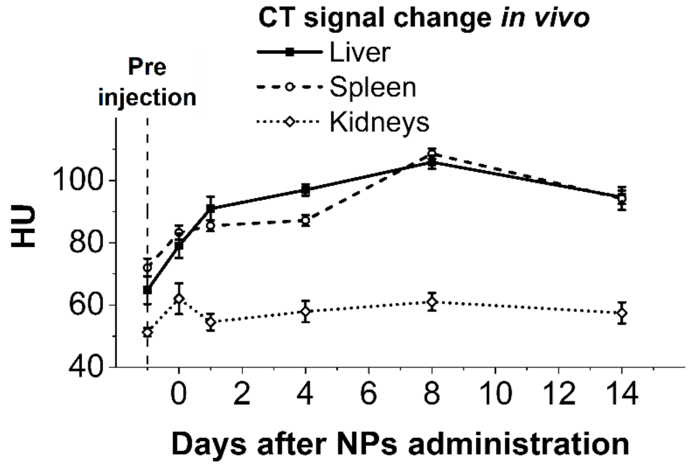

Figure S6. ***Cytotoxicity boxplots and SD-SE plots.*** Boxplots of cytocompatibility and corresponding plot of SD and SE for PEG-Au-Fe-B and reference PEG-Au NPs incubated for 24 and 48 h with BJ (A-D), PC3 (E-H) and HEK (I-L) cells at various concentrations from 5 up to 300 μg/mL.

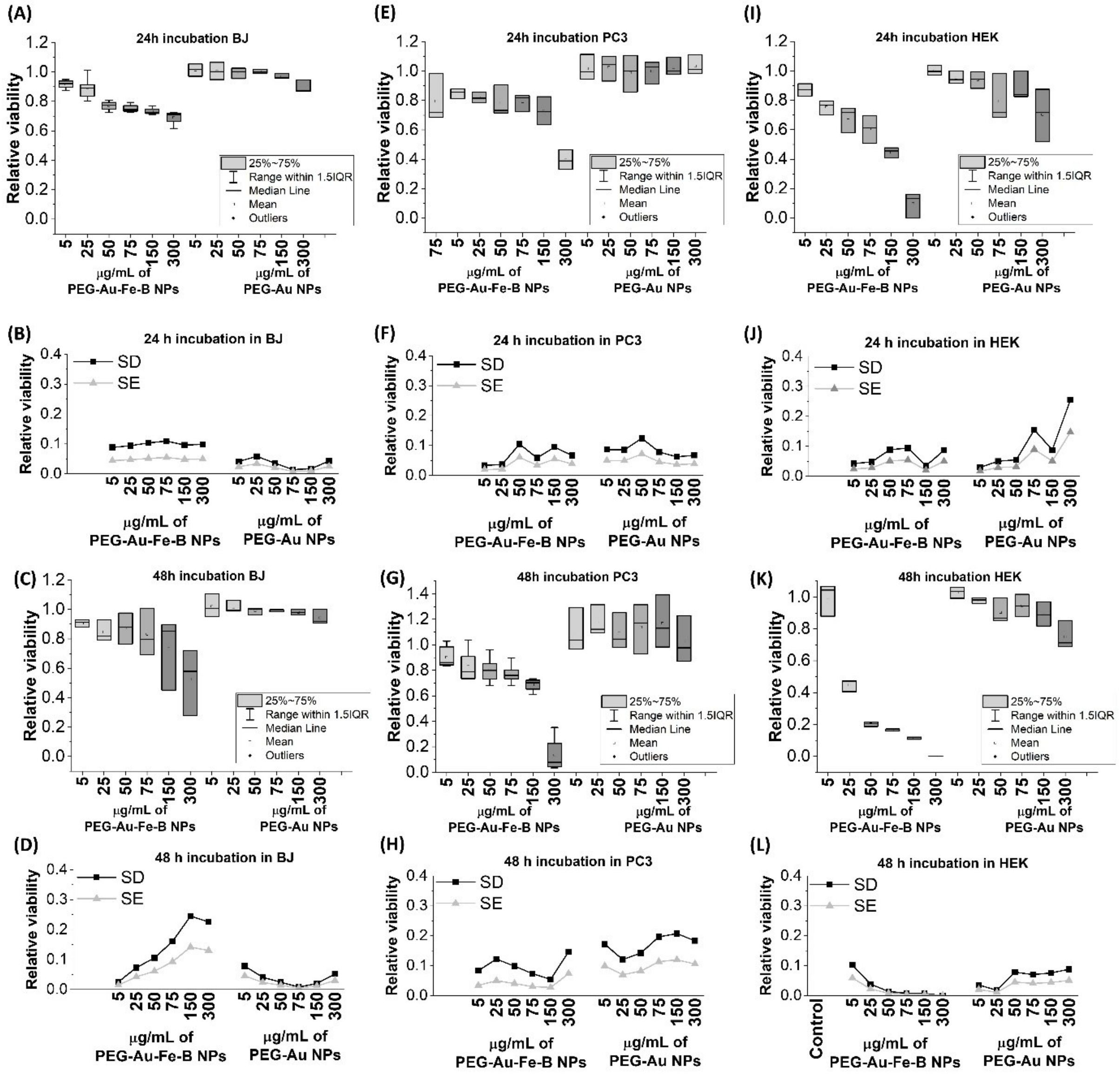

Figure S7. ***Cytotoxicity vs Au, Fe and B content in Au-Fe-B NPs.*** Cytocompatibility reported versus the individual concentration of Au, Fe and B in the PEG-Au-Fe-B and Au in reference PEG-Au NPs incubated for 24 and 48 h with BJ (A-F), PC3 (G-L) and HEK (M-R) cells at various concentrations. Determined by MTT (N = 3). The total Au-Fe-B NPs concentration is reported in Figure 5.

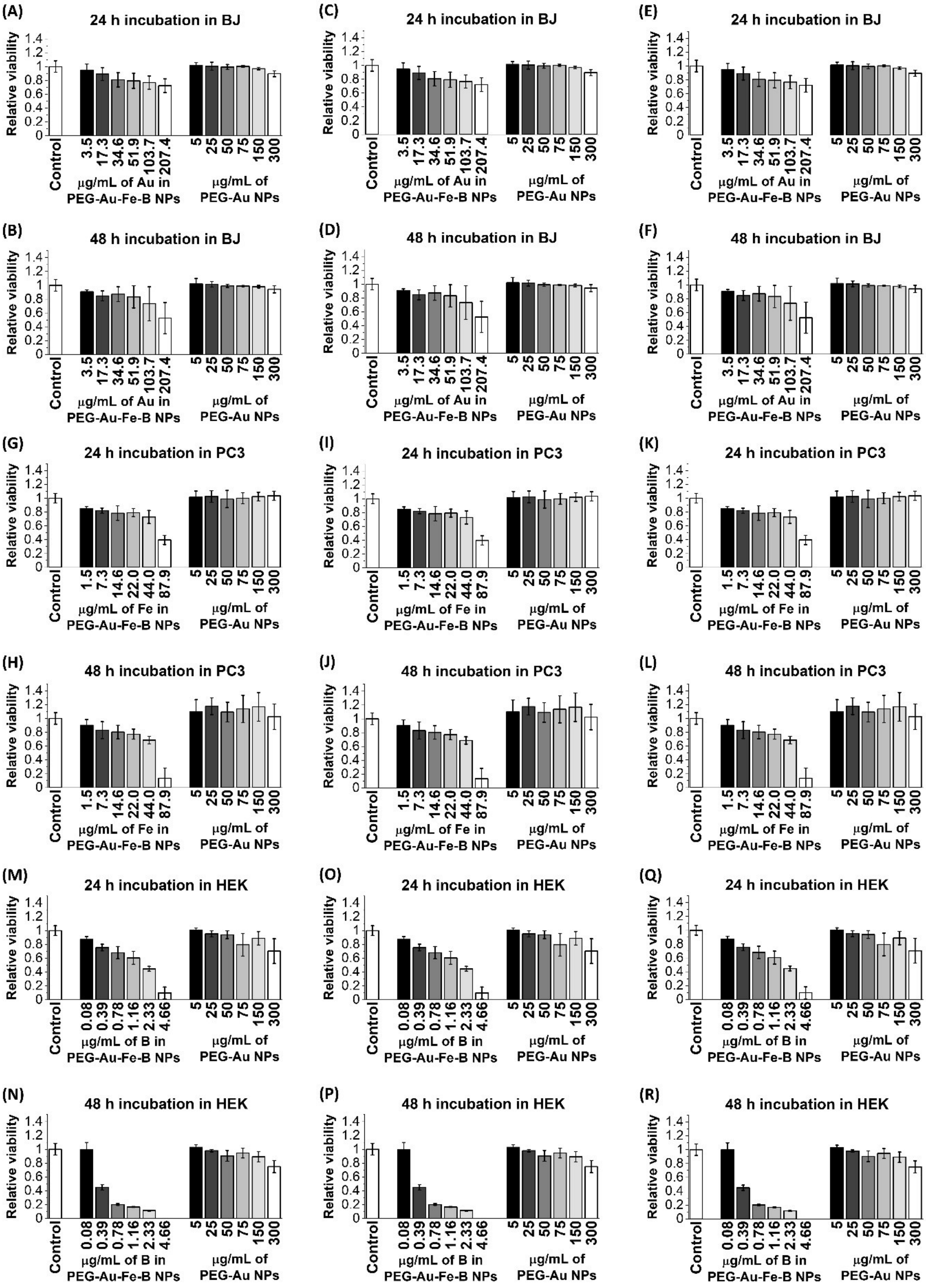

Figure S8. ***XRT clonogenic assay sketch and images.*** (A, created with biorender.com) Sketch of the clonogenic assay procedure for the experiments reported in Figure 5H and Figure 6A-B. After irradiation (1), cells were detached from the plates using a standard protocol with trypsin and each condition was counted separately using a hemocytometer. Then, each group was plated (2) in triplicate with 400 cells/well in a 6-well plate for the 0 and 2 Gy, and at 2×, 4× and 10× cell concentration for 4, 6 and 8 Gy, respectively, using 2 mL of medium per well. After 8 days in the incubator (3), the plates were coded for a blinded count, then colonies were stained after medium removal using 0.5 mL/well of a crystal violet solution (4). After staining solution removal, plates were rinsed by immersion in water and left to dry at room temperature. Colonies were then counted (5), and the relative survival fraction was calculated using the standard formula for Survival Fraction (SF) = (number of colonies)/(number of cells seeds × Plating Efficiency (PE)). The number of cell colonies, defined as groups of cells composed at least by fifty cells and visible at naked eye, was thus obtained and the graph of cell survival at different dose was built (6). The protocol aligns with those in literature.[1,2] (B) Representative images of colony formation for 6 and 8 Gy in control PC3 cells (control) and cells previously incubated with PEG-Au-Fe-B NPs for 24 h at 100 μg/mL (Au-Fe-B NPs). Well diameter is 35 mm.

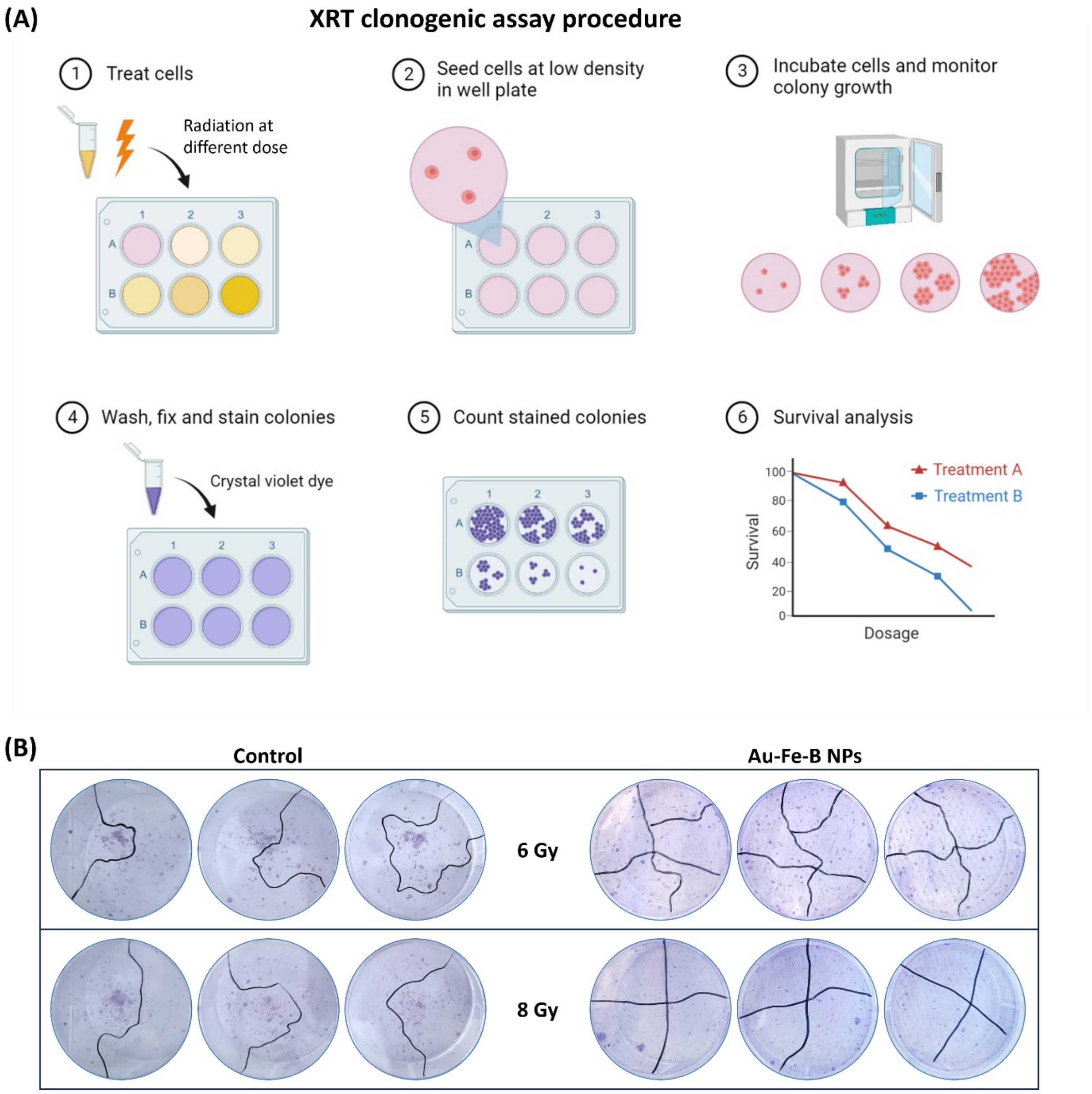

Figure S9. ***BNCT clonogenic assay sketch and images.*** (A, created with biorender.com) Sketch of the clonogenic assay procedure for the experiments reported in Figure 5H. After irradiation (1), cells were seeded at different concentration in the Petri dishes (2) and incubated for colony formation (3). Based on previous experience with *in vitro* BNCT clonogenic assays,[3] high seeding (5000 cells per Petri dish) for the highest dose and low seeding for the lowest (50 cells per Petri dish) were used. Cells were allowed to reproduce for eight days, after which they were fixed and stained (4). The cell clones grown afterwards were counted (5) both in a control Petri and in the Petri seeded with irradiated cells to provide the Plating Efficiency (PE) and the Survival Fraction (SF), respectively. The PE was used afterwards to normalize the clone number to obtain the SF due to treatment and the graph of cell survival at different dose was built (6). (B) Representative images of colony formation for 6 and 8 Gy in control PC3 cells (control) and cells previously incubated with PEG-Au-Fe-B NPs for 24 h at 100 μg/mL (Au-Fe-B NPs). Well diameter is 35 mm.

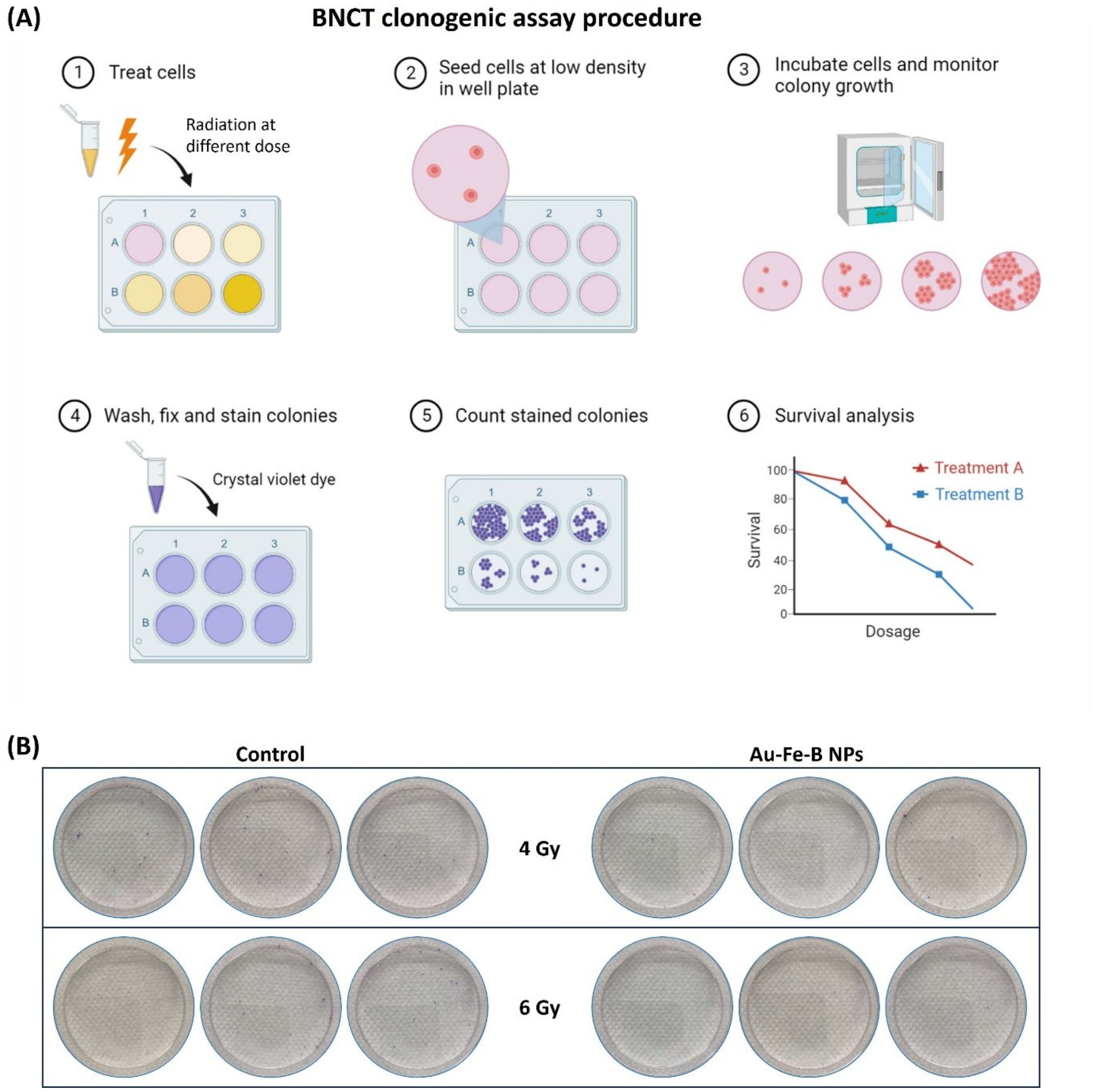

Figure S10. ***XRT clonogenic assay images with Fer-1 and Cat.*** Representative clonogenic plates of the XRT experiment at 4 Gy with Cat or Fer-1 in control PC3 cells (A, control) and cells incubated with PEG-Au-Fe-B NPs for 24 h at 50 μg/mL (B, Au-Fe-B NPs). Well diameter is 35 mm.

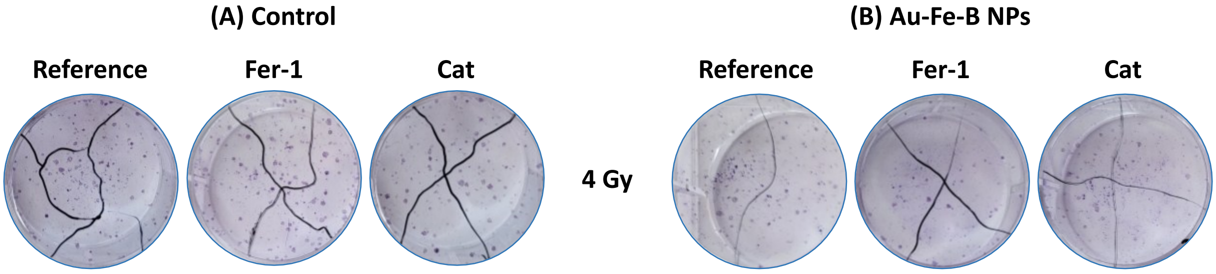

Figure S11. ***Intracellular hydrogen peroxide assay.*** For the detection of intracellular hydrogen peroxide with the ROS-Glo H_2O_2 assay, PC3 cells were treated with Au-Fe-B NPs at a final concentration of 50 µg/mL for 24 hours. Two experimental groups were analyzed: cells treated with XRT alone and cells treated with NPs + XRT at doses of 2 Gy and 4 Gy. All conditions were tested in independent triplicates. Luminescence values were expressed in relative light units (RLU) and used to compare ROS production between experimental groups.

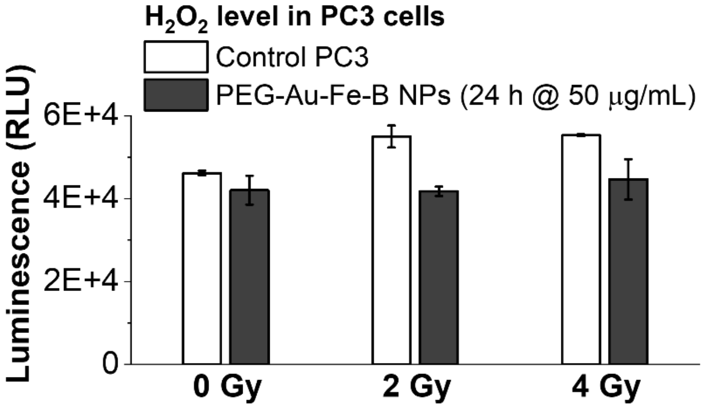

Figure S12. ***GSH/GSSG assay.*** For the GSH/GSSG assay using the GSH/GSSG Ratio Detection Assay Kit II – Fluorometric, PC3 cells were treated with Au-Fe-B NPs at a final concentration of 50 µg/mL for 24 hours. All measurements were performed in independent triplicates and results were expressed as GSH/GSSG ratios calculated from fluorescence values based on a standard curve prepared using the kit's glutathione standards.

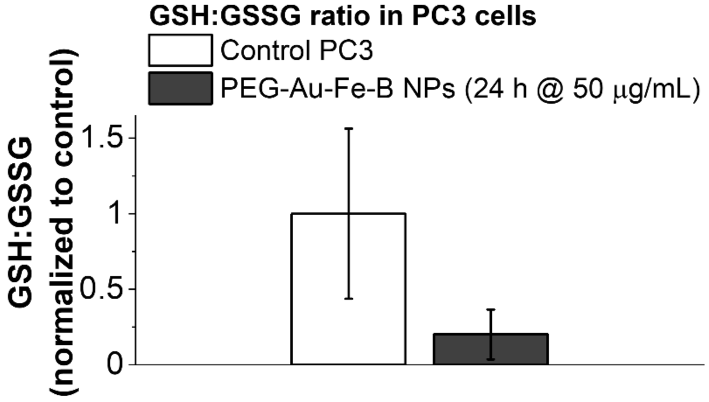

Figure S13. ***In vitro neutron autoradiography.*** Assessment of the intracellular presence of boron in PC3 cells was performed by neutron autoradiography on cells grown on a solid-state nuclear track detector (Lexan). (A) Optical microscope images (5× and 10× magnification) of control PC3 cells (no NPs) and cells treated with Au-Fe-B NPs after staining (cell image) and after etching (image of charged particle tracks). (B) 50× magnification images of the PC3 cells treated with NPs, in which the tracks are more easily identified as black pits (ca. 1 μm). These pits are clustered as expected for cells with a consistent uptake of boron-containing NPs. The image of the same ROI in transmitted light allows for identification of the tracks as white circles with a black contour.

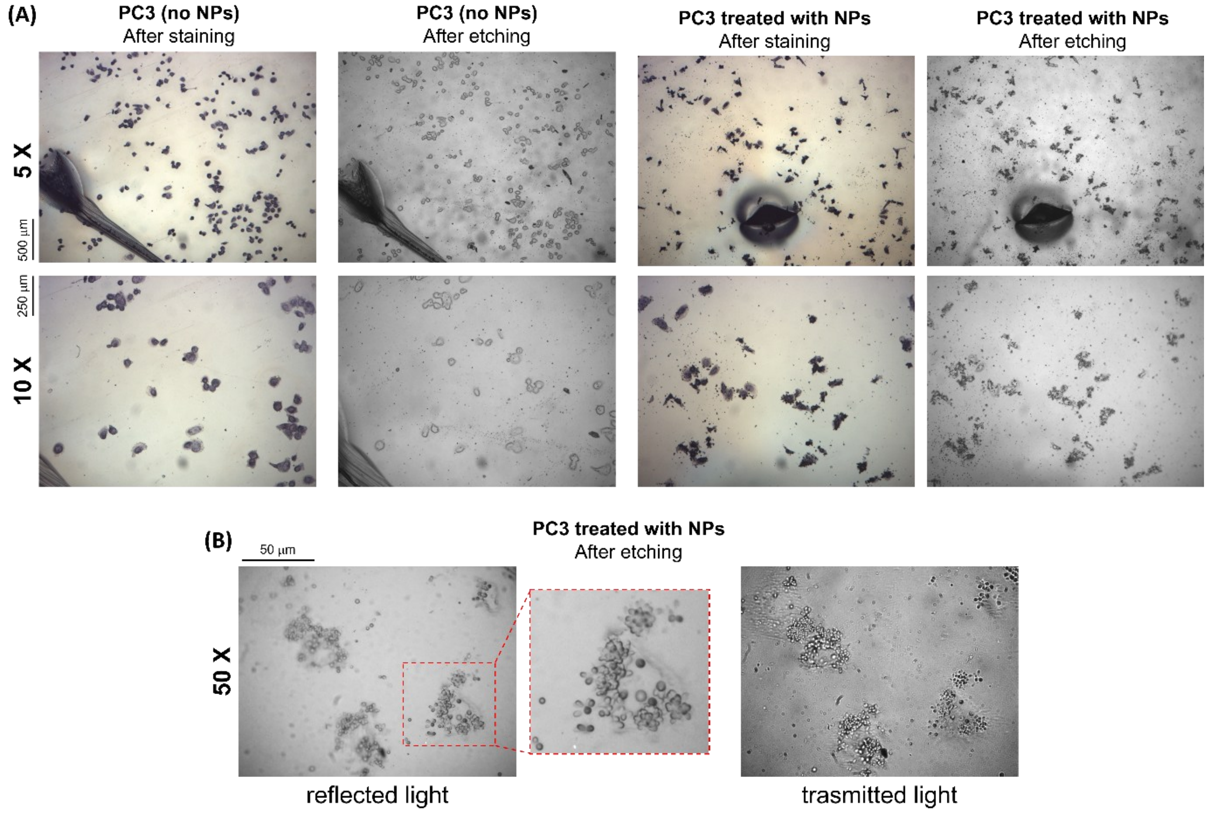

Figure S14. ***XRT in a 3D cell model.*** Efficacy of Au-Fe-B NPs for XRT of 3D cell cultures (spheroids). (A-D) Spheroids size measured at different timepoints just after the XRT treatment at 0 (control, A), 5 (B), 10 (C) and 15 Gy (D). (E-F) Spheroids size measured at 9 (E) and 11 (F) days after the XRT treatment at different doses. Error bars represent the SD of six replicates. (G) Optical microscope images showing the spheroids at 11 days after the treatment with different doses. In addition to the diverse size, spheroids morphology evidenced, in the case of the cells treated with the NPs, an irregular shape, the presence of debris and dead cells in the surrounding, more necrotic areas and an overall reduced recovery capacity of cells that, in fact, stopped their expansion.

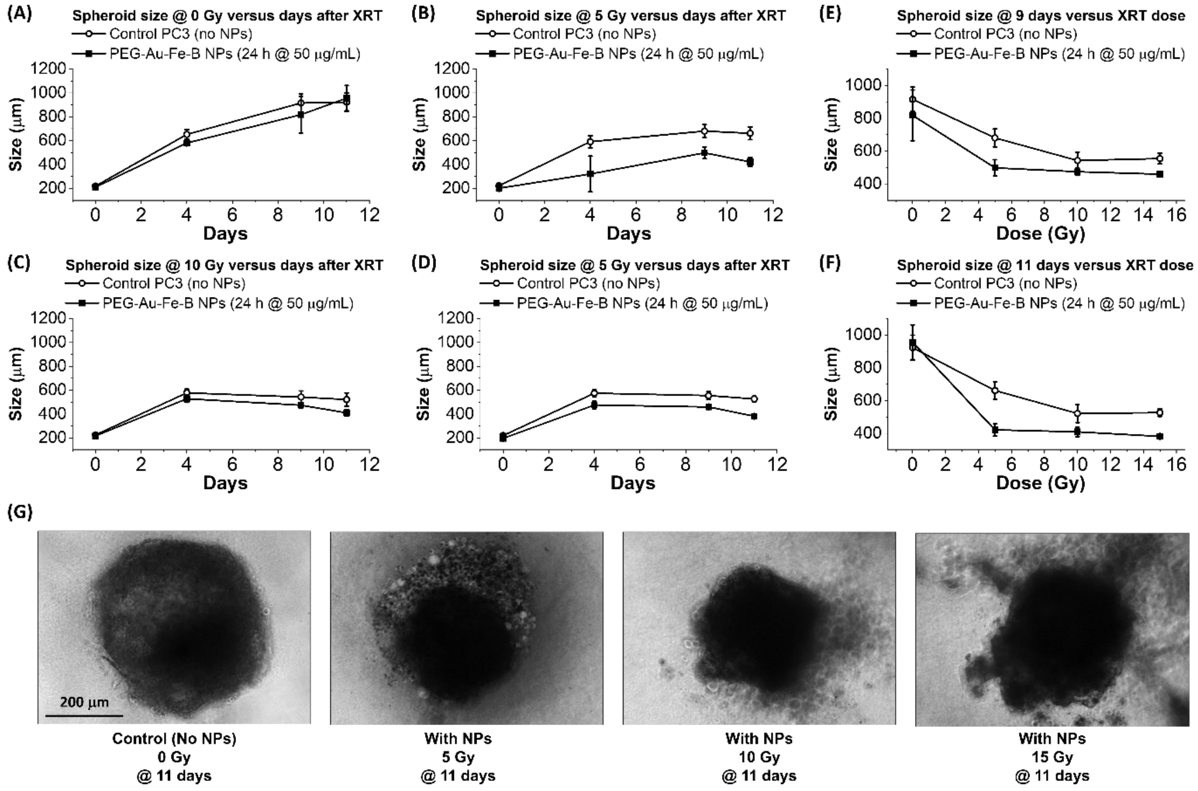

Table S2. ***Formation energies and structure of Au-B-Fe models***. Wireframe spheres are Au, green solid spheres are Fe, and pink solid spheres are B.

<table>
<tr><td colspan="2">$Au_{31}BFe$</td></tr>
<tr><td>B@Fe(T_d)
E_{form}=2.38 eV
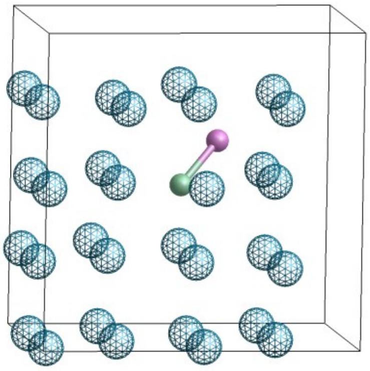</td><td>B@Au(T_d)
E_{form}=2.81 eV
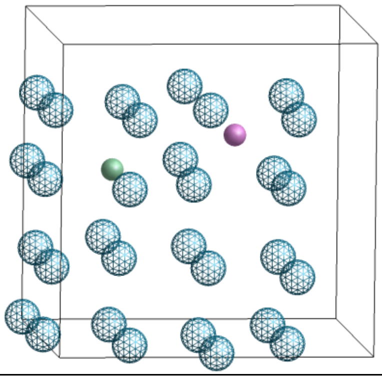</td></tr>
<tr><td>B@Fe(O_h)
E_{form}=1.73 eV
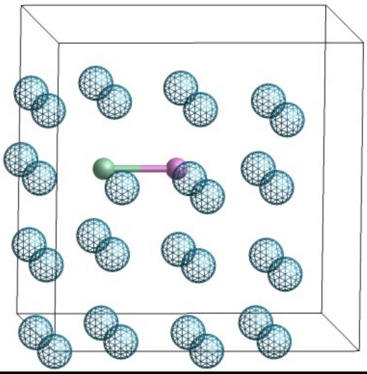</td><td>B@Au(O_h)
E_{form}=2.12eV
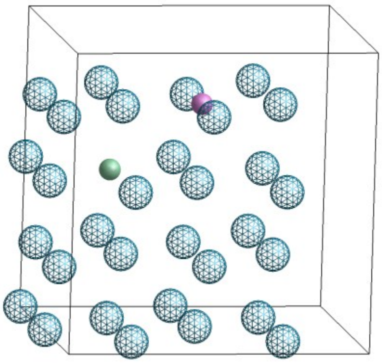</td></tr>
<tr><td colspan="2">$Au_{30}B_2Fe$</td></tr>
<tr><td>B@Fe
E_{form}=2.79 eV
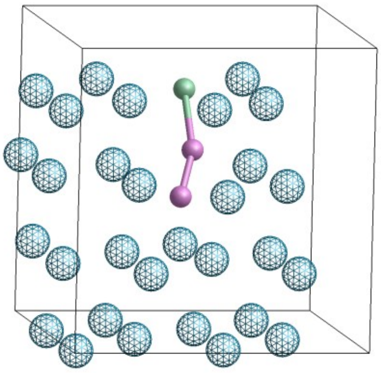</td><td>B@Au
E_{form}=3.17 eV
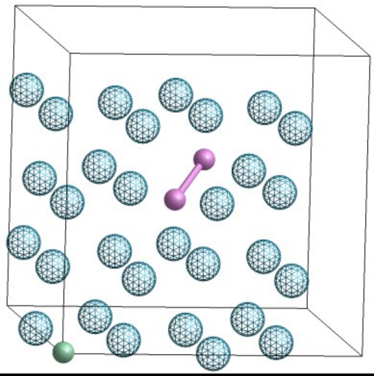</td></tr>
</table>

Table S3. ***Formation energies and structure of the $Au_{29}B_2Fe_2$ model with O.*** Wireframe spheres are Au, green solid spheres are Fe, pink solid spheres are B, red solid spheres are O. The O site (T_d or O_h) near the corresponding element (@Fe or @B) is reported in parentheses. For the O@Fe(O_h) configuration, the O relaxes to the O@Fe(T_d) configuration.

$Au_{29}B_2Fe_2$ + O	
$Au_{29}B_2Fe_2$<br>E_{form}=3.05 eV<br>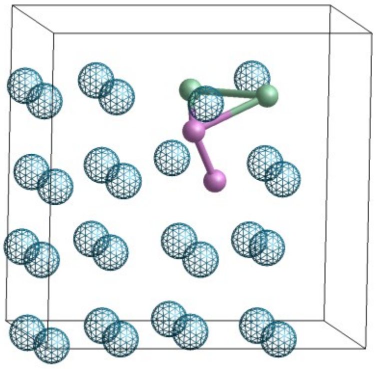	
O@B(T_d)<br>E_{form}=1.90 eV<br>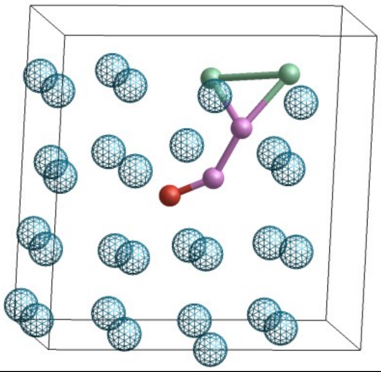	O@B(O_h)<br>E_{form}=1.21 eV<br>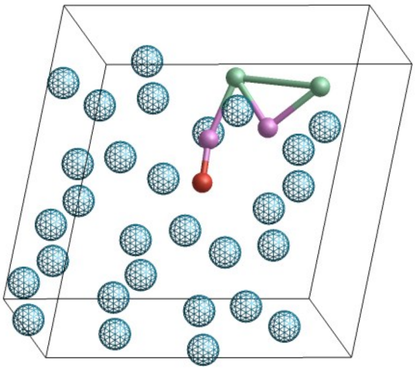
O@Fe(T_d)<br>E_{form}=2.15 eV<br>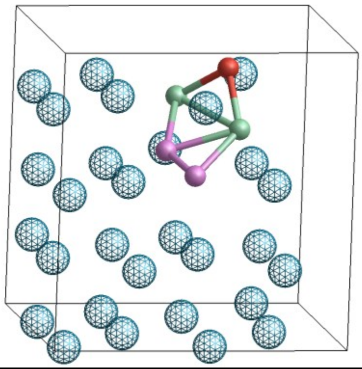	O@FeB<br>E_{form}=0.94 eV<br>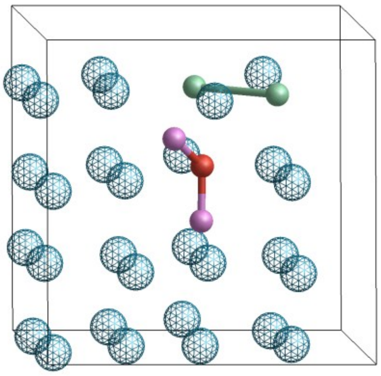

Figure S15. ***Side view of the 111 slab with Fe channel.*** Wireframe spheres are Au, solid green spheres are Fe.

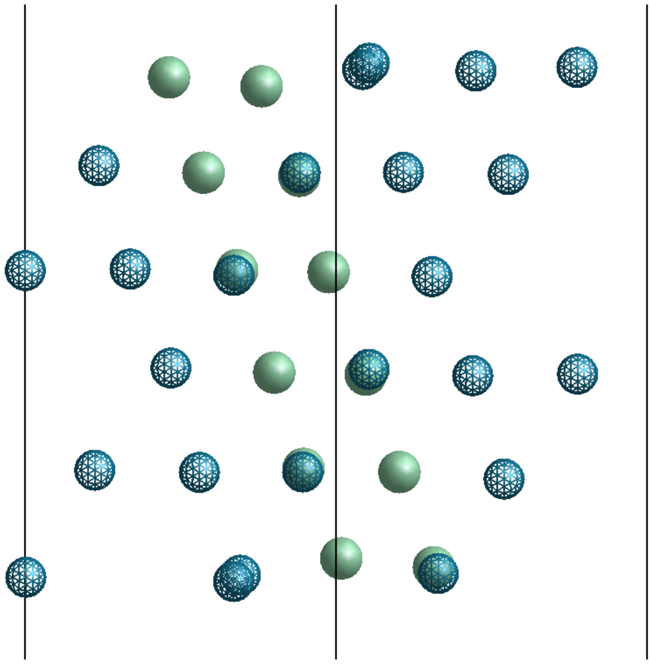

Bibliography

(1) Franken, N. A. P.; Rodermond, H. M.; Stap, J.; Haveman, J.; Bree, C. Van. Clonogenic Assay of Cells in Vitro. *Nat. Protoc.* **2006**, *1* (5), 2315–2319.

(2) Rafehi, H.; Orlowski, C.; Georgiadis, G. T.; Ververis, K.; El-Osta, A.; Karagiannis, T. C. Clonogenic Assay: Adherent Cells. *J. Vis. Exp.* **2011**, No. 49.

(3) Cansolino, L.; Clerici, A. M.; Zonta, C.; Dionigi, P.; Mazzini, G.; Di Liberto, R.; Altieri, S.; Ballarini, F.; Bortolussi, S.; Carante, M. P.; Ferrari, M.; González, S. J.; Postuma, I.; Protti, N.; Santa Cruz, G. A.; Ferrari, C. Comparative Study of the Radiobiological Effects Induced on Adherent vs Suspended Cells by BNCT, Neutrons and Gamma Rays Treatments. *Appl. Radiat. Isot.* **2015**, *106*, 226–232.
